# Ninth International Medical Congress

**Published:** 1887-10

**Authors:** 


### NINTH INTERNATIONAL MEDICAL CONGRESS.

HELD IN WASHINGTON, D.C., SEPTEMBER 5, 6, 7, 8, 9, AND 10, 1887.

(CONTINUED FROM THE SEPTEMBER NUMBER.)

### THIRD DAY.

GENERAL SESSION — WEDNESDAY, SEPTEMBER 7TH.

The Congress convened at 10 A. M., and Professor Durante, of Rome, Italy, one of the vice-presidents, was invited to occupy the chair, whilst Professor Mariano Semmola, of Naples, Italy, delivered a general address on

BACTERIOLOGY AND ITS THERAPEUTIC RELATIONS.

The object of medicine is to cure disease. To cure diseases we must know the causes that produce them. The external causes are visible and tangible, but to discover the internal, invisible causes is the aim of medical science. To solve this problem we must employ the true method of solving all problems — the experimental method. Doctors lost themselves in fantastic speculations before this method was known. The wonderful progress of physiology has been made in the light of experimental methods. When morbid conditions had been studied, instead of going on with the same careful and slow research, physicians wanted to hurry on, because they wished simply to cure the sick. To apply the experimental method and, at the same time, go fast is, in the nature of things, impossible. Thus it happened while physicians were making experiments in the laboratory, instead of having patience to master their studies, they came at once to a conclusion. New hypotheses had to be made, and without knowing it they began again the same errors that had characterized the medicine of an earlier day. New systems thus came into the field, that were the opposite of the experimental method. If medicine is to progress and be a science, it must not leave the experimental method, otherwise there will be nothing but renovations of error and loss of time.

THE ERROR OF THE DAY

is bacteriology considered as the key to all pathology. Bacteriology should be studied, because it teaches what is in the microscopical world, the existence of which we had never dreamed—a world in which man lives and which is filled with

ENEMIES OF MANKIND.

We drink millions of microbes in water, and respire millions in the air. Sometimes these microbes affect us—perhaps killing in a few hours.

When we strive to cure the sick, we must proceed cautiously, because, before there has been a careful demonstration, if we attempt to deduce a remedy, there is danger of doing harm to the sick instead of curing them. This is the great harm modern bacteriology does. Doctors concluded at once that microbes were the cause of disease, whereas, in many cases, microbes are but

EFFECTS OF DISEASE.

We ought to reproduce the disease artificially by a microbe before concluding that it is the cause. The experiments made have not given any satisfactory results, except in carbuncle and tuberculosis. To conclude hastily that this or that microbe is the cause of any disease, is but to ignore or set aside the experimental method. The demonstration which the experimental method demands in this case would be complicated, because we would not only have to know that the microbe existed, but we would have to know what was the condition of the blood necessary to the culture of that particular microbe, and science tells us that, for the present, this is a problem we cannot solve.

We know very little of the normal condition of the blood, and biological chemistry is still in its infancy. Man cannot separate himself from these millions of parasites among whom he lives. That bacteriology may be a guide in the cure of disease, we must not only learn all we can of the microbe itself, but, more important than all, must ascertain all that is possible of the conditions of the field of culture. The science of the present knows nothing of the conditions of these fields of culture in living organisms. It is thus evident that in the present condition of bacteriology it cannot be taken as a guide for the treatment of internal diseases. The older school of medicines spoke of organic dispositions, or tendency to such and such a disease. This expression had no meaning, but it expressed the fact. When bacteriology speaks of a need for a special field of culture it says the same thing, because we do not know of what the field of culture consists. Therefore, this cannot be called a science, because a science is never composed of unknown things; it goes from the known to the unknown. If a man supposes a fact instead of demonstrating it, the phenomena of nature are not reproduced. When he resorts to hypothesis the power of man disappears. If nature's laws are not respected, the telephone does not work, the electric light does not flash, the steam-engine stops. The doctor, then, is the only one who pretends to become the master of nature without knowing her laws. Referring again to the failure of medicine to follow up a discovery in the scientific way with thorough research and demonstration, and its tendency to accept conclusions quickly, Professor Semmola said that modern bacteriology may lead the way to the most fruitful field of inquiry in the future, but for the present it has produced no practical results in the cure of internal diseases. It has not, he claimed, been demonstrated in what measure microbes are the causes of diseases. He therefore hoped that the younger generation would continue ex perimental researches with the thoroughness o method which the great masters have transmitted to us. They must renounce their preconceived ideas in medicine, and interrogate nature without torturing her. Scientific independence must be preserved. They must not proceed without measuring their steps. He trusted that his desire for scientific independence in such researches would be echoed in this land of independence.

### SECTION ON GENERAL MEDICINE.

### THIRD DAY—AFTERNOON SESSION.

Professor John A. Octerlony, Louisville, Kentucky, read a paper entitled

### THE STUDY OF THE NATURAL HISTORY OF DISEASE.

He was deeply impressed with the importance of the subject in its practical bearing upon the solidity and permanency of medicine as a science. In endeavoring to discuss this he desired not to be understood as belittling in any way that science. It is not only well to admire past achievements, but also thoroughly to review the past to ascertain its deficiencies and seek the means most likely to overcome them. The study of the natural history of disease is most conducive to this end.

This subject is difficult on account of the vast scope and the complexity of disease. In looking over the ground we see in every department of pathology unmistakable evidences of the all-pervading presence of laws.

The eagerness of the physician to relieve pain and restore health often causes him to manifest distrust in nature's agency in the cure of disease. Diseases themselves are perfectly natural though not normal conditions of the living body; and the same power which called them into being may also not unreasonably be supposed to be adequate to their removal.

The truth is, nature possesses far greater power in curing disease than is admitted. A knowledge of the natural history of disease is the necessary basis upon which to estimate all medication.

The multiplicity and divergence of scientific opinions result from the neglected study and consequent ignorance of the natural history of disease.

To arrive at a proper knowledge of the natural history of disease, the co-operation of large numbers of medical men throughout the world is necessary, dissecting and attending to the various morbid conditions which affect the human race. These observations must include patients affected with various diseases modified by age, sex, occupation, etc., the duration of the malady, the events marking its course, the mortality, and mode of death. We shall then be in a position to judge with positiveness the value of a drug in shortening disease, preventing complications, or averting a fatal result. There are many obstacles to the execution of such a plan. To some it will appear culpable to withhold the assistance of art, and consign a patient to the exclusive care of nature. Independent of the consideration of the benefits to be derived therefrom, all objections to such experimentations may be fairly met by the following arguments:

First.—Since we do not hesitate to subject patients in our public hospitals to treatment with medicines the action of which is imperfectly or not at all known, and consider it legitimate, it can be no less so in similar cases to simply watch the operations of nature.

Second.—It must not be overlooked that nature, having inflicted disease, is also in many instances adequate to its cure. This is true of both light and severe diseases—those in their nature grave, such as malignant fevers, tuberculosis and cancer spontaneously recover.

It is beyond dispute that the worst forms, and apparently hopeless cases of fever, recover unaided by medicine. Recovery from tuberculosis has most frequently occurred under simply good hygienic surroundings. Hence the steadily increasing favor with which the profession regards the climatic treatment of this formidable enemy of our race.

Third.—The strong tendency to recovery in acute affections is often admitted, and is a good reason in many cases for reducing medicinal interference to a minimum, and in many cases amply justifies allowing the disease to pursue the undisturbed and natural course.

Fourth.—The character of self-limitation which we now know to characterize many diseases should be a warning to those who entertain exaggerated ideas of the results produced by their treatment, and an encouragement to those desiring to study the natural history of disease.

Fifth.—Were it not that the *vis medicatrix naturæ* is no imaginary power, but a living reality, it would be impossible to understand how it is that many quite feeble or absolutely inert medicaments could have attained such a high reputation in the treatment of various and severe affections. To-day "sage" is known to have no medicinal power, yet its history is classical, and it has received the indorsement of the learned as an effective medicinal agent. How many drugs have, like the garden sage, been lauded for imaginary virtues, but after awhile have sunk, like it, into well-merited disuse. Yet while in vogue, and medical journals were filled with accounts of their vast powers, it was the *Great Silent Mother* who wrought the cure.

Sixth.—The indisputable fact that recoveries take place from similar diseases under quite opposite plans of treatment allows no other inference than that the recoveries are due to nature alone.

Seventh.—When one recalls how marvelously patients sometimes get well under the rude and pernicious medication inflicted by quacks, one is forced to conclude that nature is not only adequate to remove the original disease, but also to overcome the artificial disease not infrequently superadded by the *energetic ignorance* of the practitioner.

He was well aware that physicians cannot if they would, and should not if they could, forego all medicinal treatment in the management of all the sick under their charge. But in private practice instances will occur when such a course would be both safe and proper.

The greatest field for observation is the hospitals. It is well to set aside a certain number of cases and compare the results of medicinal and non-medicinal treatment.

Medical students should be taught not to beieve in the exclusive power of art in the treatment of diseases, but more prominently the utility of studying their natural history.

Dr. Cronyn, of Buffalo, New York, discussed he paper. He thought that, as it is the natural h istory of disease that teaches us, attention to it is best. After all, it often depends upon nature, keeping the patient quiet and assisting nutrition. The laity think diseases due to a poison varying in degree and intensity, according to the severity of the illness.

Dr. H. B. Hemenway, of Kalamazoo, Michigan, said: Our work is not as physicists, but as doctors, teachers. I fully agree with all that has been said in the paper. We, as physicians, have made the mistake of following the popular demand "to do something." In the methods of our studies of disease we should seek to learn the cause, general progress, and termination. We ought to study to aid recovery. We can do our patients but little good in any other way.

Dr. W. J. Scott, of Cleveland, Ohio, was pleased with the paper. He believed we would have a broader foundation by studying the history of the treatment of disease—what has been done, that we may know what ought not to be done. He objected to the too free use of compendiums of medicine instead of thorough and detailed works. He had faith in medicines. He then spoke somewhat out of line with the subject of the paper, upon the increased efficacy of our materia medica and the action of drugs upon specific diseases.

The President, Professor A. B. Arnold, said: If we are called to see a patient in an attack of apoplexy, the friends invariably want the physician to do something when we know that the patient ought to be kept quiet, to allow the clot to form. There is a form of diabetes where an excess of sugar taken into the body will be eliminated by the kidneys in the urine. We know that by taking only as much sugar as can be assimilated the patient will be cured. There is another form of diabetes where the sugar is from the animal tissues, and consequently this is not improved by such treatment. These he gave as examples showing the advantage of a correct knowledge of the natural history of disease in determining treatment.

Dr. Thomas H. Manley, of New York, N. Y., believed each disease had a special course and tendency to recover. He discussed quite freely the use and abuse of drugs.

Professor Octerlony, closed the discussion by saying that he had been edified by the spontaneous expressions of regard for remedies; there was certainly not a word in his paper in reference to this subject. "I thought when Dr. Scott said so emphatically that he believed in remedies that perhaps he thought I did not." He wished to see an exact science, which could only be realized by the method suggested in the paper.

Dr. Pavy, of London, England, remarked, "I felt that I could not leave without saying I am heartily in accord with the idea that there is a natural history of disease, just as we each have a natural history of ourselves. By studying the natural history of disease we enable our own natural history to have its free play." He believed we needed, as physicians, more knowledge of agencies to cut short the natural history of disease. We also needed medicines to influence the mind as well as the body.

THE DISEASE OF INEBRIETY AND ITS TREATMENT.

Dr. T. D. Crothers, of Hartford, Connecticut, read a paper with this title. He mentioned the historic fact that inebriety was called a disease long before insanity was thought to be other than spiritual madness. On an old papyrus found in one of the tombs of Egypt, dating far back into antiquity, a clear recognition of inebriety was found. Herodotus wrote, four centuries before the Christian era, that drunkenness was both a disease of the body and mind. Other writers were noted who had urged this same idea, for centuries down to the present. By a strange shifting of events, insanity, which was supposed to be a spiritual affection until a comparatively recent date, is now studied as a physical disorder, while inebriety, which was mentioned as a disease twenty centuries ago, is still invested with the superstition of a spiritual origin.

Dr. Crothers said when inebriety is seen from a scientific point of view, it is found to be controlled by laws which vary according to certain physiological, psychological, and physical forces, of which heredity, environment, culture, nutrition, brain-and-nerve-vigor, are most prominent. When accurately recorded histories of many cases of inebriety are studied and compared, certain fixed ranges of causes appear, which follow some regular order of movement. These causes were summarized as follows:

1. Certain conditions of heredity, certain physical and psychical shocks and nerve-injuries are followed by inebriety in a large proportion of cases.

2. Certain structural changes of the brain and functional perversions, certain disturbances of nutrition and nerve-irritation, precede inebriety in many cases.

3. Certain unstable brain-organizations, regular, retarded, and defective brain-developments, etc., are apparent causes in many cases.

4. Certain diseases seem to have a special predisposition to develop into inebriety without any exciting causes.

These groups were discussed in detail.

In dipsomania and periodical inebriety a condition allied to epilepsy was mentioned. The explosions of the craze for drink were called nerve-storms, the regularity and uniformity of these periods were mentioned.

The uniformity of the symptoms of inebriety were described. Inebriety was affirmed to be increasing, and becoming more concealed every year. The coarser features were giving away to mania and suicide, etc.

The evidence of drink-cycles were described, and the temperance movements were affirmed to be reactions of the drink-cycles, and governed by laws and forces unknown.

In the treatment, work-house hospitals, conducted on a military basis, were urged, such hospitals to be built from the license fund and made like quarantine stations, where the patients could be controlled and placed in the best condition for recovery.

Different plans of treatment were mentioned in detail, and the profession urged to take up this subject and solve its problems along the line of accurately observed facts.

Professor George E. Stubbs, of Philadelphia, Pennsylvania, was reminded, by the paper, of what Professor O. W. Holmes once remarked: "That in order to treat some diseases, we needed to go back two hundred years." When we know that we are dealing with children from hereditary drunkards, we should properly advise about the bringing up of these children. He, too, had profound respect for church influences, but these children must abstain from alcoholic beverages if they are to be given the best chance of avoiding the unfortunate course of the parent.

Professor William F. Waugh, of Philadelphia, Pennsylvania, said: I believe in these remedies but for other reasons than that claimed. Inebriate asylums cure only to make the patients worse. On account of the fact that he has been kept idle, supported by kind friends, and having every attention given him, he becomes indolent and careless, desiring only to be pampered. The patients ought to be made to do physical labor. Too much is now attributed to mental influences; they also need attention given to the physical part of their being.

He had a case where a week before the attack of inebriety there would be an absence of bile in the passages.

Another case was presented to him where there was a red deposit in the urine preceding this attack. Appropriate remedies relieved these cases.

Dr. Cronyn, of Buffalo, New York, spoke of the Italian method of treatment, which consists of giving the patient food steeped in wine until he gets a strong dislike to alcoholics. He claimed that this treatment seldom failed.

Dr. Crothers replied that these cases are practically insane. There is no one plan of treatment. Inebriety ought to be considered a disease as much as typhoid fever, and we are as capable of caring for it as any clergyman can be. They are emotionally diseased, and this conversion is a part of that disease. All these methods, such as moral influences, etc., are good, but such treatment is incomplete.

Dr. Octerlony said that some of the greatest drunkards he ever knew had been clergymen, on account of which it seemed improbable that moral influences alone were sufficient for a cure.

Dr. Cisna, of Pennsylvania, then read a paper on

TYPHOID FEVER.

---

### SECTION ON THERAPEUTICS AND MATERIA MEDICA.

WEDNESDAY, SEPTEMBER 7TH—THIRD DAY.

The first paper read was on

### RHAMNUS PURSHIANUS.

Dr. John E. Brackett, of Washington, D. C., gave a botanical and chemical description of rhamnus purshianus, or cascara segrada. He considered the fluid extract, given twice daily in doses of five to fifteen minims, to be curative in chronic constipation, but the conditions favoring the occurrence of the constipation should also be corrected.

Dr. Murrell, of London, England, did not consider that the claims for special virtues in this drug had been well supported. He did not regard it as superior to the other varieties of rhamnus.

Dr. Phillips had obtained such results in chronic constipation that he had been much pleased with it. He had found in it points of superiority to both senna and rhubarb.

Professor F. Woodbury, of Philadelphia, Pennsylvania, said that in many cases of hæmorrhoids accompanying constipation he had found both conditions disappear simultaneously under the effects of cascara, especially in cases of women, who are liable to suffer with these disorders.

Dr. Ralph Stockman, of Edinburgh, Scotland, read a paper entitled

### ON THE PHARMACOLOGY OF SOME BODIES DERIVED FROM MORPHINE.

The experiments were conducted jointly by D. B. Dott, F.R.S.E., and Ralph Stockman, M.D. The relationship between chemical constitution and physiological action must always be a subject of deep interest to pharmacologists. In this short paper they proposed to mention briefly the changes in action resulting from various modifications in the constitution of morphine.

It was found by How, in 1854, that by acting on morphine with methyl iodide, a body was obtained which he named hydriodate of ethylmorphia, and which he regarded as a substitution product, the methyl iodide being supposed by him to replace one of the hydrogen atoms in morphine. In the light of our present chemical knowledge we regard How's body as an addition product, iodide of methyl ($CH_3I$) being simply tacked on to the morphine molecule.

In 1869, Crum Brown and Fraser investigated this body among others, and showed that the original action of morphine is quite lost and a curare action substituted in its stead. From their nomenclature there can be no donbt that these observers regarded such bodies as addition and not as substitution compounds. Notwithstanding this, we find that in all text-books and reference-books these substances are always named and described as if they were substitution and not addition bodies. It is at present generally held that the substitution of methyl for hydrogen in alkaloid causes the latter to act like curare, no matter what its original action may have been. The addition products, however, are very different in action from the substitution products.

The formula of morphine is $C_{17}H_{17}NO_3(OH)_2$ and it contains, therefore, two molecules of hydroxyl. It is the hydroxyl-hydrogen atoms which are replaced most easily by alcohol radicals. With regard to the action of morphine, it may be divided into two stages—(1) narcosis succeeded by (2) tetanus. Methylmorphine, $C_{17}H_{18}(CH_3)NO_3$, is morphine in which one H has been replaced by $CH_3$. Codeine has the same constitution, and this body is simply codeine prepared artificially from morphine. The action is exactly the same as that of codeine derived directly from opium.

Ethylmorphine, $C_{17}H_{18}(C_2H_5)NO_3$, has the same action exactly as methylmorphine.

Acetyl morphine, $C_{17}H_{18}(C_2H_3O)NO_3$, and diacetylmorphine, $C_{17}H_{18}(C_2H_3O)_2NO_3$, have a similar action to the two preceding bodies. That is, they produce narcosis in very small doses, which is followed by tetanus when larger doses are given. All these bodies are much more active than morphine, and smaller doses are required. In dogs, however, they produce much greater distress and much more marked vomiting and diarrhœa. From our examination of their action, we think that none of them are in a position to replace morphine clinically.

Dr. George S. Hull, of Pennsylvania, inquired if the author could explain why the acetyl compound exerted such exaggerated effects as compared with the methylmorphine.

Dr. Stockman said that he could not explain this at present, but that he hoped to do so in the future, as these investigations are still in progress.

Dr. Murrell spoke of the usefulness of apomorphine as a prompt emetic. The rule laid down in the British Pharmacopœia to prepare the solution extemporaneously he regarded as puerile. The solution changes color, but retains its physiological effects for years. He had tried apocodeine, but had not been satisfied with the results and had gone back to apomorphine. In regard to codeine, he regarded it as less satisfactory than morphine and its employment in diabetes as merely due to fashion, and that the profession will eventually go back to morphine.

Dr. Stockman: Codeine is really the more poisonous of the two, although it is usually given in much larger doses than morphine is.

Dr. Brackett believed that codeine was less irritating to the stomach than morphine, and he was pleased with it in cough mixtures, in cases where morphine could not be given.

Dr. A. L. A. Toboldt, of Philadelphia, Pennsylvania, read a paper on

CARLSBAD MINERAL WATERS.

In cases of chronic hypochondriasis he obtained remarkable results from the use of the imported Carlsbad waters. The salts obtained by crystallization were less efficient, but the quell-salz or sprudel-salz powder, given in aerated or plain water, were found to be a good substitute. Mild cases of diabetes mellitus were entirely relieved. One case of obesity lost twenty-six pounds in less than four weeks under the use of the salt. The improvement in this case was permanent.

Dr. George S. Hull, of Chambersburg, Pennsylvania, read a paper on

SO CALLED ANTISEPTIC ACTION OF CALOMEL WHEN GIVEN IN LARGE DOSES.

In cases of dysentery most prompt results in relieving tormina and changing the character of the stools were manifested. It acts as a cholagogue, producing a free flow of bile, sweeping out the contents of the bowel, and as a mercurial it exerts an antiseptic action. In cholera its usefulness in large doses has been advocated by some and denied by others.

The relative value of large and small doses of calomel were freely discussed by the members of the section.

In closing the discussion Dr. Hull said that he was dealing with epidemic dysentery and not with ordinary dysenteric conditions. The first effect of the calomel is to empty the bile-ducts and get a large flow of bile, and relieve the congestion of the liver and intestinal vessels. The second effect is due to the solution of the mercurial in bile, as pointed out by Headland, and this probably acts as an antiseptic in the bowels. The object is not simply to purge the patient, but to obtain a copious bilious discharge from the action on the liver. Where the tongue was most coated he got the best results. He believed that patients are killed by the use of opium and astringents.

### SECTION ON ANATOMY.

### THIRD DAY—MORNING SESSION.

Dr. Joseph N. Dickson, of Pittsburgh, Pennsylvania, then read a paper entitled

ANATOMICAL CONSIDERATIONS IN REGARD TO THE AMPUTATION AND DISARTICULATION AT THE ANKLE-JOINT, BY A NEW METHOD.

This method was first made public in 1875, and later in 1881. An incision should be made in advance of the line laid down by Syme, around the sole of the foot from malleolus to malleolus, and also an incision a little above the plane of the articular face of the tibia. The disarticulation was carried out by division of the ligaments of the ankle-joint, and the dissection was begun from above downward and backward, keeping the lateral section in advance of the posterior one. As the knife was carried backward and downward it included the periosteum and bursa between the tendo Achillis and the head of the os calcis. The advantages of this operation are a long plantar flap, shortening of the superior section, division of the malleoli obliquely, and, as stated above, preservation of the bursa between the tendo Achillis and the head of the os calcis. His methods of dressing were also simple. The oblique division of the malleoli allowed a better adaptation of an artificial foot and leg. Of fifty-two cases three deaths occurred, one or two of which were complicated by other injuries. The average stay in hospital was twenty-one days, and this included patients from four to sixty-five years of age.

### AFTERNOON SESSION.

Dr. H. C. Boenning, of Philadelphia, Pennsylvania, read a paper regarding

DESTRUCTION OF THE DISSECTING-ROOM OFFAL, in which he spoke of the difficulties of disposing of the waste anatomical material. He enumerated the most usual way of getting rid of this offal by burial, storage, cremation, by alkalies, by mincing it and throwing it in the sewers, by giving it to the buzzards, and by boxing it up and sending it away. He found great objections to all these methods on account of expense and trouble. He thought he had found the solution of this unpleasant problem by the use of the Gregory furnace, the principles of which were so simple and cost of construction so slight. He found that the small cost of fuel and the small amount of ash left, and the entire absence of foul gas, rendered it an indispensable accessory for every school of anatomy. The furnace was lined with asbestos upon the firebrick.

Dr. Boenning further reported a curious

### ANATOMICAL ANOMALY.

In the subject, a colored female, aged thirty, was found an entire absence of the arch of the aorta. There were two vessels going from the left ventricle, the one up to supply the head, neck and upper extremities, and the other down to become the thoracic and abdominal aorta. In other respects the subject was normal.

---

### SECTION ON OPHTHALMOLOGY.

### THIRD DAY—MORNING SESSION.

Professor F. C. Hotz, of Chicago, Illinois, read a paper entitled

### RESTORING THE NORMAL POSITION OF THE FREE TARSAL BORDER IN TRICHIASIS.

The essayist began by the introduction of a few anatomical facts bearing upon the subject. He showed that the free edge of the tarsal cartilage in the normal eyelid is placed at an angle of ninety degrees to the surface of the eyeball, and that the eyelashes are inserted at an angle of about ninety degrees to the free border of the cartilage. The angle made by the hairs with the free border may be considerably altered, as in blepharitis ciliaris, without the hairs touching the cornea or surface of the globe. But if the free border of the cartilage is turned inward by shortening of the conjunctival surface of the cartilage from shrinkage caused by inflammation, then it is easy to see how the hairs will sweep over the cornea. This may be remedied either by lengthening the posterior or shortening the anterior surface of the cartilage. It is not necessary, nor does it occur that the curve of the cartilage is changed. Lengthening the posterior surface is impracticable, but it is proposed to show how the position of the free edge can be restored by shortening the anterior surface. An incision is made just below the upper border of the tarsal cartilage, the lid being stretched downward by an assistant, and the skin and muscular tissue dissected off from the cartilage down to the roots of the cilia. Then an incision is made directly back through the cartilage to the conjunctiva, met by another starting from a line about two millimetres above, and the wedge-shaped piece removed. A narrow ribbon of skin being now cut from the edge of the flap, it is replaced, and the sutures carried in through the edge of the flap, then through the upper border of the cartilage and out through the upper margin of the incision in the lid. The result of this procedure is apparent, the apposition of the edges of the wedge-shaped wound of the cartilage is good without sutures, and the normal appearance of the lid restored. To avoid cutting through the conjunctiva when making the incision through the cartilage the tactile sense of the finger placed on the inner side of the conjunctiva at the free border is sufficient, but a button-hole at this point does no harm, and does not affect the result of the healing.

Professor S. J. Jones, of Chicago, Illinois, said the difficulty of treating these cases of trichiases satisfactorily, and the importance of doing so, had been made manifest in the many different operations which had from time to time been devised for relief from the affection. Nearly all of them gave some temporary relief, but it is desirable to secure more lasting results.

In estimating the advantages to result from the operation as advocated by Professor Hotz, the risks to the integrity of the eye-lid from the extensive dissection must be considered, as well as the cosmetic effect after cicatrization.

From the statement made that the operation may be repeated with safety, two conclusions may naturally be drawn, viz.: That the primary operation may also be performed with safety, and that it is to be understood that it becomes necessary to repeat the operation. As to permanency of relief obtained by this operation, which is one of the most important considerations in connection with all the operations for the remedy of the affection, Professor Jones stated that several of the cases, which had previously been operated upon by Professor Hotz by this method, either as originally proposed or as modified by him recently, had subsequently been under Professor Jones' care, in which the relief at first obtained had not continued. These patients had been seen after intervals varying from a few months to a year or two. Either these must have been exceptional cases, or this mode of operating does not seem to afford results that are much more satisfactory than others obtained from the various methods of operating in the past.

Professor Frothingham, of Ann Arbor, Michigan, thought that Professor Hotz's operation will give better results than anything we now have.

The principal trouble will be in reducing the relaxation of tissue.

Dr. J. L. Thompson, of Indianapolis, Indiana, said that while country doctors could not muster as many cataracts as some of our foreign brethren, they had an abundance of this variety of trouble. He thought that no one operation would ever be found suitable for all these cases, and that procedure must be modified to suit circumstances.

Professor P. D. Keyser, of Philadelphia, Pennsylvania, had had considerable success by removing the entire cartilage.

The President said that Dr. Hotz had apparently dropped the idea of curving out the cartilage, and he wished to know the reason. The reply was that the idea of the incurvation of the cartilage had been found to be incorrect, and that therefore there was no need to alter the curve.

Dr. B. Pitts, of St. Joseph, Missouri, next read a paper on

THE BEST METHOD OF OPERATING FOR ENTROPION.

His paper was an exposition of the advantages of electrolysis, not only in removing the troublesome hairs which had assumed faulty position, but also in producing resolution and absorption of the thickened and infiltrated tissues, and in this way, even when relief was only partial, preparing the way for the easier performance of blepharoplastic operations.

Dr. W. F. Holcombe, of New York, N. Y., spoke of a method which he had put into practice, of placing the hairs in position, turned away from the cornea, and holding them in that position by silk surgeon's plaster. He had invariably had excellent results, and renewed the application from time to time, as required.

Dr. E. Landolt, of Paris, France, read a paper on

OPERATION FOR STRABISMUS.

He called attention to the marked peculiarities distinguishing this from all other operations on the eye, because it always concerns both eyes. Squint is always binocular. A man with one eye does not squint. Cyclops has no squint. Cataract and other operations may be done without taking into consideration the condition of the other eye, but not so with strabismus-operations. He dwelt upon the importance of the precautions to be taken for the success of the operation, and the causes of convergence and divergence. A most powerful aid in the correction of squint is the desire for binocular vision. Without binocular vision there is only an apparent cure, a cosmetic effect, because it leaves out the most powerful aid in the cure of strabismus. It is necessary before operation to determine the nature and measure, the degree of the refraction and accommodation, to find how far vision has been lost, and to remedy it if possible. The agents for this purpose are atropia, glasses, cessation from work, and orthoptic exercises. He never operates without satisfying himself that he has got the full effect of non-surgical measures. If the correction is made in the child while young and without these precautions, divergence may occur later. The difficulty of determining beforehand just how much to do to get a certain effect was remarked, and that it was better to do too much than too little. It is easier to diminish than to increase the effect. Dr. Landolt never operates on two homonymous muscles at once. He rather does a tenotomy of one, and an advancement of its antagonist. The remedies for over-effect in operations for convergent squint are stoppage of atropia, removal of stitches from advanced muscle, and use of the other eye. No case should lose its power of convergence or divergence after the operation, as without that power binocular vision would be impossible. If divergence persist, advance the tenotomized muscle. This can easily be done to the tenth or twelfth day.

In divergent squint, on the other hand, the danger is in doing too much. If the divergence is of long standing and the eye amblyopic, then tenotomize with advancement, or do two tenotomies. Do not use atropine, but exercise the eye in the required direction. He often prefers advancement to tenotomy to remedy faulty position. It is more complicated but not more dangerous, and has a greater and more favorable effect than tenotomy. The operation for strabismus is not to be considered as a cure, but only as an adjunct to orthoptic treatment.

---

THIRD DAY—AFTERNOON SESSION.

Dr. George F. Stevens, of New York, N. Y. read a paper on

SOME IMPORTANT PROBLEMS RESPECTING INSUFFICIENCY OF THE OCULAR MUSCLES.

He said that anomal.es of the ocular muscles were of as frequent occurrence as those of refraction and accommodation, while the literature of the subject was very scanty, and what existed would lead one to infer that only one of the muscles was liable to insufficiency—the internus—while the deviations in other directions had been ignored or barely mentioned.

The object of the paper was stated to be to suggest certain problems, rather than to explain apparent contradictions. For instance, a case showed with a prism over one eye, insufficiency of the externi of 8° at twenty feet, and at one foot insufficiency of the interni of 10°. There was an adducting power of 50°. Now, there could not be an insufficiency of both interni and externi at the same time. Another case, with the prism eliminated from the question, had diplopia, homonymous of 4° at twenty feet, and crossed of 5° at two feet. Here seems to be a contradiction of results and a problem to explain.

Dr. Stevens then entered into an explanation of the terms which he used in designating the muscular variations. He uses Orthophoria to express the correct equable balance; Heterophoria to express any departure from it. He divides Heterophoria into Esophoria, a tending of the visual lines inward; Exophoria, a tending outward; and Hyperphoria, a tendency of one image to rise above the other. Latent heterophoria is as common as errors of refraction are, but is often overlooked, and is much more difficult to discover. In case hyperphoria exists, in what way are we to determine in which muscle the fault lies? Again, how shall we determine the absolute amount of anomaly and the best manner of procedure? Dr. Stevens then detailed his own mode of examination, always prescribing prisms of a degree somewhat less than the hyperphoria. He believed it was better to correct too little than too much. He does not believe that prisms are curative or permanently beneficial, but that they are of much use in diagnosis.

Professor J. F. Fulton, of St. Paul, Minnesota, in a paper, entitled the

ADVANTAGES OF EARLY OPERATION IN STRABISMUS, spoke of the difficulty of overcoming diplopia or amblyopia where they exist after operation. He agreed with Soelberg Wells, that supression of the image results in amblyopia from disuse, where the trouble occurs in children, and even in cases where the difficulty is of a later date. He favors operating in children at an early date. If operation is not admissible, then the other eye should be covered, and the squinting eye exercised carefully at short intervals. Amblyopia met with in strabismus is either primary or secondary. If primary and congenital nothing can be done, but he thought many cases were secondary and due to the same cause as the squint. The vision of the defective eye sometimes rapidly deteriorates when the image is suppressed. A case in point was one where V. $=\frac{20}{20}$ in the right eye, and $\frac{20}{30}$ in the left, raised to normal by a suitable glass. Some time after the fixing eye was lost by accident, when the left eye was found to have only $\frac{20}{80}$. This was raised, after use for some time, to $\frac{20}{30}$. Other cases were quoted, of two members of the same family, one of whom was operated on, with the result of improving the vision from $\frac{20}{40}$ to $\frac{20}{30}$, while in the other, which was let alone, the degree of strabismus remained stationary, but the amblyopia increased.

Dr. C. H. Abadie, of Paris, France, read a paper on

FAULTY EYE MOVEMENTS AND THE MEANS OF REMEDYING THEM.

He reviewed the modes of diagnosis, particularly in relation to the differentiation between those cases that require complete and partial tenotomy, and dwelt on the ability to control the amount of correction by removing more or less of the muscular fibers. It is easy to change a partial tenotomy into a complete one, if it be necessary.

Dr. E. O. Shakespeare, of Philadelphia, Pennsylvania, read a paper entitled

ON THE STRENGTH OF THE SUPERIOR RECTI MUSCLES AS A CAUSE OF ASTHENOPIA.

He found many cases of asthenopia not relieved by correction of the refraction, and external and internal recti in which there was want of proper action of the superior or inferior recti. In testing, we may find that the displacement of the image is greater when a prism is placed over one eye than when over the other. If a correcting prism be used it will be found that one superior rectus is stronger than the other. To correct the muscular error, if there is an error of refraction, he decenters the lenses, and if no refractive error is present, then he puts on prisms.

Dr. Landolt, of Paris, France, gave a demonstration of his method of strabometry, and showed how easily the patient and tapes were placed in position and the accuracy of the result. The tapes are made by Dubois, rue Monsieur le Prince, Paris, France.

Dr. H. Power, of London, England, after stating that it was not so long since the operation was done, like dividing a tendon in orthopedy, said that he is not now so certain as formerly of a positive cure. A great deal of care is now taken of the cases before operating, and even with it all, we will have cases that appear to be just as bad after operation as before.

Dr. Shakespeare rose to ask about the successive removal of muscular fibers by Dr. Abadie in small degrees of insufficiency. He wished to ascertain something of the permanency of the relief afforded.

Dr. Landolt, in replying in the absence of Dr. Abadie, said that he could not answer that question, as he had never done that operation, and probably never would do it. He always divided the whole tendon. He believed that Dr. Abadie claimed a very good and durable effect.

Dr. G. S. Norton, of New York, N. Y., asked if Dr. Stevens would tell what was the matter where both external and internal insufficiency appeared to exist, without hyperphoria. The doctor replied that that was just one of the problems he had submitted to the section, and that he did not know.

Dr. J. A. White, of Richmond, Virginia, spoke of the great value of Dr. Landolt's paper, and added a few remarks on his own results in advancement of the capsule, with which he had obtained as good results in cases of considerable degree as by advancement of the muscle.

Dr. Thompson, of Indianapolis, Indiana, was afraid that after all a large proportion of cases return to their original condition.

The president submitted a question as to the time for operation.

Professor D. S. Reynolds, of Louisville, Kentucky, suggests the exercise of the retina with strong lenses, even where considerable amblyopia exists, with the hope of improving vision before operating.

Dr. B. J. Baldwin, of Montgomery, Alabama, is satisfied that there are many cases in which amblyopia may be prevented by an early operation. A year or two ago he believed that amblyopia was congenital, but he does so no longer.

Dr. Landolt, in closing, thanked the section for their kindness and indulgence, and expressed his pleasure at the free discussion which had been drawn out.

Dr. A. G. Heyl, of Philadelphia, Pennsylvania, then addressed the section on

ABNORMALITIES OF THE VISUAL AXIS.

He said that the definition of the visual axis is involved in obscurity, and is as well a matter of extreme importance. He reviewed the terms and definitions at present used, showing their incorrectness, as Gesichtslinie, Blicklinie, and their mechanical and not physiological basis. Collimating axis is better, but it belongs essentially to monocular vision, in which one eye dominates over the other.

His true definition is a line from the macula, directed directly in front when the eye muscles are in a state of rest or equilibrium. It is a conception low down in consciousness, as is the conception of the median plane.

Through the pull of the tendons on the sclera, the recti muscles keep the macula pointing straight ahead. He then entered into the subject of the origin of the macula—whether from the fœtal cleft, which would call for a rotation of at least ninety degrees, which is said to have been observed. Then he says: "My position, for want of a better, is that the pull of the recti, antagonized by the ciliary muscle, is the factor in the production of the macula."

Dr. Ole Bull, of Christiania, Sweden, wished to know how Dr. Heyl accounted for the existing of two maculæ in some birds with restricted vision.

Dr. Heyl, in closing, in answer to Dr. Bull, stated that he knew that those cases existed, but that he would have to examine the arrangement of the muscles before being ready to explain it. He thought the same process of tension might take place in two different directions.

### SECTION ON OTOLOGY.

### THIRD DAY.

Dr. R. Tilley, of Chicago, Illinois, read a paper on

INHERITED SYPHILIS AS A FACTOR IN SUPPURATIVE INFLAMMATION OF THE MIDDLE EAR.

He expressed surprise at the marked absence of reference to this subject in general syphilography and otological literature. Politzer, in his text-book, makes no allusion whatever to inherited syphilis as a possible cause of suppurative inflammation of the middle ear. Dr. Albert H. Buck does not even mention, under this head, inherited syphilis in his book on "Diagnosis and Treatment of Ear Diseases." Keyes, Woods' library edition, refers to a middle-ear affection from primary syphilis, but makes no reference to middle-ear affections from the inherited affection. Dr. St. John Roosa does not refer to it. Dr. Thomas Barr, in an indefinite way, says: "As in nearly all the diseases of the ear hereditary tendency plays an important part in the causation." There is, however, no specification as to the character of the hereditary tendency. Diday says: "Cases of suppurative otitis are also seen associated with this type of disease, but it is open to some doubt if it is dependent directly on syphilis." Sturgis, in his American edition, says: "The lesions of the ear are even yet but little understood, and are nearly all confined to deafness which comes on suddenly and are frequently unaffected by treatment." Sir W. B. Dalby ignores the influence of inherited syphilis in middle-ear affections. In Ziemssen's Cyclopedia, however, occurs the following: "This is the starting point of catarrh of the tympanum, and a frequent source of deafness and tinnitus aurum. . . . These conditions are especially apt to occur in consequence of inherited syphilis."

A general comparison was made with the cornea and the membrana tympani, and special attention drawn to the universally recognized relation of inherited syphilis and interstitial keratitis, and the claim made on theoretical grounds that the tissues of the membrana tympani might be expected to suffer correspondingly with the cornea.

A comparison was also made with mucous membrane of the nose and that of the middle ear; and the peculiar symptoms given by Trosseau as characteristic of inherited syphilis, as it manifests itself in the mucous membrane of the nose, were supposed to find their counterpart in the ear.

Reference was made to the relative mildness of the secondary and tertiary manifestations of syphilis, and it was supposed that suppurative discharge from the middle ear should be less frequent in the negro if the influence of hereditary syphilis played an important part in the white races.

The practical observation of the author could furnish cases, but the reports were not given on the supposition that the question being fairly presented to the profession, nothing but their own observation could produce the conviction necessary for daily practice.

The author concluded by stating that his general practice in determining the presumptive course of a case of suppurative inflammation of the middle ear is to cleanse thoroughly the affected organ and note the appearance of the tissues, to inspect the nasal and pharyngeal spaces, the teeth, the scalp, the cervical glands, and if the cervical glands are enlarged, the teeth irregular and decayed; if the features are puny and shriveled, the hair thin and dry, pustules in the scalp, hypersecretion with swollen mucous membrane in the nose and a special predisposition to disturbance of the alimentary canal, if he does not conclude that the patient is the subject of inherited syphilis, he does conclude that he will thrive better, and the suppurative middle ear will heal more effectually by the use of what are known as anti-syphilitic remedies.

A paper was read by Dr. J. Baratoux, of Paris, France, on

### CHANGES IN THE INTERNAL EAR IN HEREDITARY SYPHILIS.

During the last few years much attention has been paid to the changes developed in the internal ear in syphilis. It has thus been recognized that the walls of the vestibule, the semicircular canals, the lamina spiralis, and the cochlea may be affected with periostitis; that the soft parts of the labyrinth become implicated with round cells; that the auditory nerve has been greatly modified in structure, and finally, that the membranes of the internal ear have been injected and bathed in a sero-sanguinolent fluid which had taken the place of the lymph. He said, I have myself, in a work on syphilis of the ear, called attention to the inflammation of the vascular loops of the external walls of the canal of corti on a level with the spiral angle and of those which run along the vasilar membrane. I have also demonstrated on several preparations a dilatation and even a rupture of the walls of the vessels.

But among the new-born, afflicted with hereditary syphilis, the changes in the internal ear have been but little studied. As a fact Hutchinson attributes the associated deafness in such cases to a lesion of nervous origin. Lancereaux and St. John Roosa hold the same opinion. Wreden refers the lesion to a gummous degeneration of the auditory nerve. Hinton, however, demonstrated congestion of the vestibule.

In a series of autopsies, partly made at the Hospice des Enfants-arnistes, and partly in the obstetric department of the Hospital Cochin and St. Louis in Paris, I have found on different occasions that the internal ear was the seat of important primitive lesions, or arising secondary from lesions of the middle ear.

Among the numerous autopsies which I have made I present only the report of forty-three cases. All these forty-three cases were affected with hereditary syphilis, demonstrated either by lesions on different parts of the body, or by lesions on the parents, which lesions had manifested themselves in the year previous to the birth of the child.

Thus there was demonstrated twenty-seven true lesions of the middle ear, four times lesions of the labyrinth, and twelve times lesions of both parts.

The object of the communication is to call attention to the changes in the internal ear.

When the internal ear was affected simultaneously with the middle ear, without pus being found in the labyrinth, the walls of the ampullæ and cochlea were reddened; the axis of the cochlea injected and infiltrated with round cells, and the parts bathed in a sero-sanguinolent fluid which had taken the place of the lymph. When the internal ear alone was affected, the blood vessels were found to be dilated, the walls thickened, and in some instances hæmorrhagic spots were found. Demonstrations of these conditions were made to the physicians associated with the hospitals whence the cases were taken.

#### DISCUSSION.

Professor G. E. Frothingham, of Ann Arbor, Michigan, thinks the papers of Drs. Tilley and Baratoux are valuable productions, since they deal with a subject hardly if at all, discussed in the works on aural affections. To cite his own custom, for example, inquiry as to hereditary syphilis in ear diseases did not receive the attention it deserves from otologists. While acquired syphilis is duly considered, the pains of investigating the possible influence of hereditary syphilis in the etiology and progress of certain aural affections, are seldom taken. Of course, when other well-marked symptoms of inherited taint are present, they are generally weighed, and the case is treated on the general principle that any constitutional disease should be cured, if possible, as a means of combating any local expression. It is not his custom, however, to attempt any special inquiry in this direction, and he does not think many others do. He readily conceives that inherited taint might give no other manifestation than, perhaps, general debility, which might be attributed to other causes. Happily, the custom of giving tonics, and alteratives as well, in the treatment of aural affections, compensated, somewhat, for this neglect, since cod-liver oil and the iodides, administered for other reasons, answered the best purpose in such inherited disease when conjoined with proper out-door exercise, bathing, cutaneous friction, and nutritious food—elements which entered into the therapeutics of most such cases. Dr. Baratoux is entitled to the thanks of the profession for his careful researches in this field; and Dr. Tilley, as well, for calling attention to the subject. He thinks it will be wise to give heed to the facts they have presented, and to remember them in examinations and the treatment of ear affections.

---

### SECTION ON DERMATOLOGY AND SYPHILOGRAPHY.

### Third Day.

#### LUPUS ERYTHEMATOSUS.

The session opened with a paper on the above subject by Dr. A. Ravogli, of Cincinnati, Ohio, In his opening remarks the author said that he would always prefer to treat lupus vulgaris than lupus erythematosus, so difficult was it to obtain satisfactory results. Kaposi had regarded the condition as a neoplasm. Hebra first described it as a seborrhœa, but modified the term by adding to it *congestiva.* All authors mention erythema and congestion as important features. Specimens were exhibited, which showed enlargement of the epithelial cells and hypertrophy of the papillæ in the stroma of the corium, and an infiltration of inflammatory cells in the meshes of the tissues, and some cells are seen between the fibers of connective tissue surrounding the hair-follicles. There is an increase in the number of connective-tissue corpuscles. The elastic fibers are swollen and enlarged, and the loculi, formed by the fibers of connective tissue, contain fluid. The blood-vessels are filled with blood. The condition is then one, not of neoplasm, but is an inflammatory process. Each layer of the skin is affected. After a patch of lupus erythematosus has disappeared there is left a thin, white, parchment-like condition of the skin resembling a scar.

There is a true atrophy of the skin, caused by pressure producing obliteration of the glands which first take part in the hypertrophy. The process is then first an hypertrophy of the histological elements, followed by an atrophy.

The cause of the atrophy is found in the blood-vessels being closed and obliterated by pressure. The primary cause consists in an irritation and stimulation of the nerves, which increases the blood-supply and causes a disturbance in the biological activity of the cells.

The author has examined scales from three cases of lupus erythematosus. The epidermic cells were found enormously enlarged, and contained a large number of round bodies which he regarded as micrococci. They formed groups and colonies, and chemical tests appeared to confirm the opinion that they were such.

Sections of the skin were made and showed the existence of the cocci in the papillary layer, most abundant where exudation has taken place, and small colonies in the interior of the fibers and in the capillary blood-vessels.

These bodies answered to the test laid down by Friedländer for micro-organisms, and Dr. Ravogli does not doubt that micro-organisms exist in lupus erythematosus, not only upon the surface, but also within the structure of the skin. He has been unable to make any culture experiments. He regards these organisms as the cause of the disease, and instead of classing it as a neoplasm, thinks it should find its place among the infectious diseases.

The inflammatory symptoms and hypertrophy are explained by the irritation of the micrococci. The function of the sebaceous glands is increased by the irritation, hence the seborrhœa and the formation of the greasy scales. This explains lupus erythematosus discroides; in the form called aggregatus the micrococci still better explain the condition.

As regards treatment, internal remedies are useless, as a rule. The application of emplastrum hydrargyrum has been considered the best treatment. The benefit of this powerful microbe destroyer strengthens the theory. When the skin is destroyed with caustics we succeed in destroying the cocci. When the sharp spoon was used to scrape the patches, or they were burned, the disease often returned in the neighborhood of the patch. Dr. Ravogli has obtained complete recovery in three cases of the disease by the use of ichthyol. Diminution in the secretion of the sebaceous glands is soon noticed. The reducing action of this agent is displayed on the underlying tissues. Ung. diachyl. hebræ goes well with ichthyol (ten per cent.), producing elevation of the epidermis and suppuration. The ichthyol is diminished to three per centum, and only slight scars result. He finishes treatment with ichthyol in collodion. Dr. Ravogli showed microscopic specimens to illustrate the pathological changes and the bodies which he regarded as cocci.

In the discussion, Dr. Knaggs, of England, asked if the reader considered the micrococci as causative, and whether the ichthyol destroyed them and the tissues, too, or acted as an antiseptic.

Dr. Ravogli replied in the affirmative. The ichthyol acted, he thought, by abstracting oxygen, upon which the life of the cocci depended.

Dr. Unna, of Hamburg, Germany, regarded the most important part of the paper that relating to micro-organisms, and he considered it the weak point as well. The cocci must be seen in all parts of the thickness of the skin, and, he believed, in the sweat-glands, too. He thought micro-organisms probably existed in the disease, and would some day be discovered, but he could not regard the specimens presented as showing them, and hoped Dr. Ravogli would continue his investigations and make cultures. He asked if the sections were made deep enough to show the sweat-glands.

Dr. Ravogli said he had not paid special attention to these glands.

Dr. Unna was glad to hear of the good influence of ichthyol, never having used it alone in this disease, but with the addition of salicylic acid. He has found resorcin beneficial and most useful as a varnish or collodion dressing.

Dr. Thin, of London, England, said that the refractive particles seen under the microscope might be micro-organisms, and might be other bodies. More characteristic stainings would be necessary and sections carried into the deeper parts, showing the same bodies before they could be regarded as micro-organisms. Furthermore, cultures and inoculations would be necessary to prove the case. Dr. Thin spoke of several rare cases he had recently seen in England, which, from their peculiar appearance, had been given the name of the cock's-comb disease, as similar cases had not been observed there. The feature of the affection was a raised, bright-red, uneven surface, with abrupt margins. He had examined specimens of the tissue, and had found it identical with that of lupus erythematosus. There was a greater degree of vascularity than usual, the epidermis was raised, and a dense mass of small cells was found between the rete mucosum and strata lucidum. The affection was found very unsatisfactory as regarded treatment. He related one case of lupus erythematosus in which an inexplicable spontaneous cure had taken place while the patient was upon an exclusive milk diet prescribed for another disease. He believed in a micro-organism, but thought it had not yet been found.

Dr. Klotz was glad to hear Dr. Ravogli say he avoided stronger and caustic remedies. He preferred salicylic acid combined with soap-plaster. He thought the discovery of micro-organisms would bring lupus erythematosus into closer relationship with lupus vulgaris, from which it had been too far alienated.

Dr. J. Zeisler, of Chicago, Illinois, thought the micro-organism theory a very plausible one; still, it was only a theory. To prove them it would be necessary to make cultures and inoculations. He did not regard the studies in this direction as entirely new, as Morrison, of Baltimore, had made similar investigations. Lupus erythematosus of the mucous membrane is an extremely rare condition. He had observed it in Vienna, in a case in which the mucous membrane of the mouth was affected. It presented a whitish condition of the membrane, the tissues were hardened, and it proved very obstinate under treatment. Caustic nitrate of silver was alone found to influence the condition. He agreed with Dr. Unna as to the value of resorcin, and that ichthyol had little effect. A ten per centum solution of resorcin in collodion had acted well.

Professor Robinson, the president, spoke especially in regard to the microscopic specimens. He agreed with Thin and Unna that in all probability a micro-organism exists, but he does not think that Dr. Ravogli has pursued proper methods, nor do the specimens show the bodies to be cocci. It is not clear that they are not extraneous matter. We must exercise great care in placing importance upon micro-organisms found upon the surface. He urged Dr. Ravogli to follow up his investigations and thought something might come of it.

In closing, Dr. Ravogli said that he labored under difficulties in making his demonstrations at this time. He considered the test with caustic potash a convincing one, as it never attacked micro-organisms, but did attack everything else. He was glad to hear the opinions of so many that micro-organisms probably existed. He would not say positively at the present time that they were causative, but would wait until he had been able to make cultures, etc., as suggested.

A paper on

ERYTHEMATOUS LUPUS OF THE HANDS, was read by Dr. Ohmann-Dumesnil, of St. Louis, Missouri, in which he says, few cases of erythematous lupus of the hands have been observed or accurately described. Existing upon the hands alone the disease is extremely rare. Treatment is sought usually on account of the appearance. He related the history of a case in which the lesions appeared upon the hands alone, had existed for a long time, and had been treated by caustic applications without much benefit. He employed concentrated lactic acid in the form of a paste, which produced severe pain, and was followed by a slough; pyrogallic acid was alone used, but the case passed from observation before a cure was effected. The author has collected forty-five cases. In twelve the disease began in the face, the hands being subsequently affected. The lesions do not extend beyond the nails and the dorsal surface is the seat of predilection. The patients were usually in good health. Dr. Dumesnil had endeavored to find cocci in the scales and sections of lupus erythematosus, but had not been successful. There is at first an inflammation of the epithelial layer, which subsequently dips down into the hair follicles, following and affecting the epithelial structures, and only extends to the dermic structures by contact.

A paper entitled

A CONTRIBUTION TO THE KNOWLEDGE OF IMPETIGO HERPETIFORMIS (HEBRA).

was presented by Dr. Josef Zeisler, of Chicago, Illinois. He first gave a short historical review of the literature of the subject, and paid especial attention to the work of Duhring, who, as he thought, went too far in including a disease like impetigo herpetiformis, so sharply characterized by its clinical course, the unmistakable efflorescences, the almost exclusive occurrence in women, in connection with the puerperium, the fatal end, etc, with other diseases under one name, that are so entirely different and mostly of a benign character. He referred to a recent paper of Kaposi, who, in describing the disease, again warns against confusion in studying a question which needs so very much to be cleared up. The author then furnished an exhaustive description of a case of this rather rare affection.

In the discussion which followed, Dr. Unna related a case which resembled both impetigo herpetiformis and dermatitis herpetiformis, but differed from both and was probably neither. It was similar, as regarded treatment, in that iodine in abundance was the only treatment which gave good results.

It resembled more an impetigo, because the blebs appearing did not from the first contain pus, as is necessary to establish the diagnosis.

---

### SECTION ON CLIMATOLOGY AND DEMOGRAPHY.

### Third Day.

Dr. A. Tucker Wise, of Engadine, Switzerland, presented a paper on

THE CLIMATE OF THE SWISS ALPS, WITH PULMONARY CASES TREATED AT AN ALTITUDE OF SIX THOUSAND FEET.

The marked peculiarities of Alpine winter climate may be enumerated as, dryness of the air and freedom from micro-organisms, mechanical irritants, and noxious gases, low temperature, plenty of sunlight, low pressure, and ozoniferous atmosphere. The results of these peculiarities upon pulmonary complaints may be stated thus:

1. By breathing aseptic air free from dust, irritation, or, perhaps, recurrence of infection by microbes in the respiratory tract is greatly lessened.

2. Vaporization of morbid secretions in the lungs takes place, promoted by reduced barometric pressure and dryness.

3. Increased oxidation of blood and tissue from sunlight, cold air, and reduced pressure.

4. Increased quantity of blood circulating in the lungs, the freedom of the circulation being aided by extended chest movements.

5. Increased activity in the pulmonary lymphatics, and a general improvement in nutrition and glandular secretion; also an exhilarating effect upon the nervous system.

The four principal health resorts of the Grisons, in Switzerland, are Maloja, Wiesen, Davos, and St. Moritz. The climates of these resorts are stimulating and tonic.

Twenty-three cases are related, showing great improvement during a residence of from one to sixteen months.

A paper by Dr. John D. McDonald, F.R.S., Inspector General Royal Navy of Great Britain, on

GROUND-AIR IN ITS HYGIENIC RELATIONS, pointed out the importance of the study of the atmosphere of the soil, but contained no especially new points of view.

Dr. P. H. Boyce, of Toronto, Canada, Secretary of the Provincial Board of Health of Ontario, read a paper upon

HOUSE ATMOSPHERES, OR ARTIFICIAL CLIMATES.

The points considered were the constituents of house atmospheres, their temperature and humidity, and air currents; the effects of house atmospheres on populations, and remedies for existing evils connected with house atmospheres.

With reference to the constituents of house atmospheres, the observations of Miquel, Koch, Aitken, and Tyndall upon indoor and outdoor air were quoted. Considerable attention was given to temperature and humidity in connection with house air.

The remedies for the evils mentioned are sunlight in abundance, greater care in the construction of dwellings, foundations, and plumbing appliances, improved municipal sanitation, and the attainment of equable heating and thorough ventilation. In conclusion, it must be recognized, regarding the fatal effects of the imperfect conditions of human life under which Indians, negroes, and many of the people of limited means exist, demand the earnest consideration of all workers in the field of climatology and demography; and since the occupations, urban residence, and limited means make it impossible for an increasing proportion of our population to enjoy the health-giving influences of rural residence and the stimulating effects of life by the ever-restless ocean, or upon the mountain side, we shall best conceive the duties assigned to us, of making it possible for every willing citizen to so live under his own roof as to maintain a vigor unimpaired for the discharge of the work lying nearest him, and to transmit to the race that is to be a legacy of physical health.

### SECTION ON DENTAL AND ORAL SURGERY.

THIRD DAY—MORNING SESSION.

Dr. Pradère, of Lyons, France, read a paper on

PHTHISIS CURED BY THE CONTINUOUS APPLICATION OF MEDICINE TO THE PALATE.

Immediately after the paper was read, Dr. James Trueman, of Philadelphia, Pennsylvania, moved that it should not be accepted by the section, but should be referred, without discussion, to Section I., in General Medicine.

A number of gentlemen gave clinics in the treatment of diseased conditions of the oral cavities, and others demonstrated their methods of filling teeth and constructing artificial dentures for patients. These clinics are spoken of as a successful feature of this section.

Dr. Metnitz, of Vienna, Austria, read a paper on

OSTEOMYELITIS.

which consisted chiefly of a report of two cases from practice.

The history of the first case was as follows: In October, 1886, a lady, aged forty-three, had two teeth extracted. A few days later she suffered with chills, which were followed by slight mental disturbances. The seventh day the patient became unconscious, in which condition she was brought to the hospital. Examination revealed that there was a large swelling over the left cheek, extending to the temporal region ; the skin covering this swelling was tense and pale in color; the sclerotic was highly colored (yellow), and the skin showed yellow tinge; the pupils were without reaction. The odor of the breath gave evidence of necrosis. The submaxillary glands were very much enlarged, and the neighboring tissues infiltrated. There was unconscious urination and defecation. Death occurred the following day. The post-mortem examination showed the membranes of the brain to be thickened and traversed by numerous vessels.' The left hemisphere was covered by a layer of pus, and the right hemisphere showed considerable pus along the track of the vessels as well as several pus-depots. The brain-substance was quite soft. The examination of the oral cavity disclosed that of the two teeth extracted the upper alveolus had almost entirely filled up with healthy granulations, whereas the lower was filled with pus. The mucous membrane in the region of this diseased alveolus was very much discolored and could easily be removed in pieces. The probe discovered nothing but dead bone. All the muscles of the neck which are attached to the left side of the lower jaw were infiltrated with pus. The periosteum was separated from the left side of body and ramus of the jaw. The alveolus of the extracted wisdom-tooth communicated by two good-sized openings with the marrow-cavity, and the marrow itself was discolored and infiltrated with fat. The cause of this extensive destructive action is no doubt to be looked for in the unclean condition of the alveolus after the extraction. Sections of the jaw show that the medullary canal was very much enlarged.

Kocher, Rosenbach, and Busch, in experimenting on animals, have found that it is impossible to produce an acute pus-forming osteomyelitis either through traumatic injury or chemical and mechanical irritation, but that such a condition can readily be brought about by infecting the fresh wound in the bone by any decaying substance.

The second case was one of multiple osteomyelitis. The patient, male, aged seventeen, suffered from an attack of osteomyelitis of the humerus, the ulna, and the lower jaw. According to Billroth, it is not settled whether this condition (multiple osteomyelitis) is due to septic influences acting on various places at the same time, or whether the infection dates from one point.

Death in this case, as in the first, was directly due to acute suppurative meningitis. When we have to deal with a simple inflammation, energetic antiseptic treatment will prove quite sufficient. In severer cases of osteomyelitis Billroth advises that the seat of disease be reached as soon as possible—the pus evacuated, the cavity thoroughly disinfected, and dressed with antiseptic dressing. Many cases present no actual deposits of pus, or abscesses, but simply an infiltration of the marrow. In such cases Billroth holds it of little value to open into the medullary canal. Neither does he advocate disarticulation or resection, because, in the first place, the exact extent of the disease cannot be foretold, and, secondly, the medullary substance of a patient suffering from osteomyelitis is in such a susceptible condition that a new injury would almost certainly prove fatal.

Dr. Jenison, of Minneapolis, Minnesota, read a paper on

ART IN DENTISTRY.

The essayist advocated the restoration in gold of all teeth that had been destroyed by caries, thereby improving both their usefulness and beauty.

In constructing artificial dentures more time should be given to the restoration of the features of the patient, and for that purpose single and not section teeth should be used.

---

THIRD DAY—AFTERNOON SESSION.

Dr. R. R. Andrews, of Cambridge, Massachusetts, read a paper on

THE ORIGIN OF THE DENTAL FIBRIL, ILLUSTRATED BY AID OF STEREOPTICON.

Dr. Andrews described his process of preparing and mounting the specimens for the microscope, which differed in no essential respect from the latest methods employed by others for that purpose.

In speaking of the formation of the fibrils, the essayist says there are two kinds of odontoblasts —those which are square toward the dentine, and others, just by the sides of the first mentioned, which are pear-shaped. From these latter, and not from the first (or square end ones), originate the dental fibril.

The stereopticon views presented showed very clearly with what patience, earnestness and intelligence the essayist worked to establish his view of the question.

Dr. Frank Abbott, of New York, N. Y., in opening the discussion, paid a high tribute to the reader of the paper for the hard work done in behalf of his specialty. In order to understand the process by which the dental fibril is produced, it is necessary for us to consider the matter from the third to the fifth month of intra-uterine life, at which period of the existence of the fœtus the papilla of teeth are so far developed that a material change is observed to be taking place. The papilla is a mass of myxomytous tissue, liberally supplied with medullary elements. In some instances at three months, at others as late as the fifth of intra-uterine life, a coalescing of several of the medullary corpuscles into one may be observed upon the periphery of the papilla adjacent to the enamel organ, which at this period may be observed forming a cap upon the papilla. The united medullary corpuscles are known as odontoblasts. The impression has generally prevailed among histologists and embryologists, that the odontoblasts were directly formed into dentine. This theory, through recent researches, has been proven to be incorrect. The odontoblasts, when viewed with a power of 1,200, show a delicate reticulum, which unites the nuclei with the walls of each corpuscle and with each other. This reticulum, as well as the walls of the odontoblasts, are the living matter which remains as the living portion of the dentine. Before the beginning of the deposition of lime salts, the odontoblasts are reconverted into medullary substance. As such they receive the calcareous basis-substance, and thus a certain territory of the papilla becomes dentine. While this process of calcification is going on, another row of odontoblasts makes its appearance, from the sides and ends of which prolongations of the living matter may be seen running into the canaliculi of the dentine already formed. A spindle or pear-shaped odontoblast, gives off one, while those with broad ends give off two, three, and even five prolongations. If the views advanced in the paper were correct, it would necessarily follow that territories of considerable size would be left in the dentine with no canaliculi whatever; nor is there any provision for furnishing these territories with any living tissue.

Dr. Fletcher, of Cincinnati, Ohio, read a paper on

PROTECTIVE DENTINE; ILLUSTRATED BY STEREOPTICON.

This paper, from its practical aspect, was of special interest to the section. The slides which were thrown on the screen showed the different kinds of protective dentine, and the essayist gave his views of how these different efforts on the part of nature to protect herself are brought about.

Dr. W. X. Suttuth, of Philadelphia, Pennsylvania, agreed with the essayist in the practical conclusions drawn; he supplemented the reader's remarks by stating that the odontoblasts remain after the development of the dentine, but can be stimulated to produce or perform their function of forming protective dentine.

Dr. J. Howard Mummery, of London, England, exhibited

PHOTO-MICROGRAPHS OF ALL THE STRUCTURES OF THE TOOTH, and explained the best method of producing them.

---

### SECTION ON GYNÆCOLOGY.

### Third Day—Morning Session.

Dr. Ernest W. Cushing, of Boston, Massachusetts, read a paper entitled

CANCEROUS DEGENERATION OF THE HYPERPLASTIC GLANDS OF THE CERVIX UTERI.

Ruge and Veit have described a condition of the glands which they considered to be in itself the nature of a cancer—a transition from innocent to malignant formation. This seems to me much less clearly demonstrable than the views which they maintain concerning erosion.

Briefly, they attribute the greater import to a certain filling up of the lamina of the glands with epithelial cells, either columnar or flat. The theory of Veit and Ruge agrees so thoroughly with Theirsch and Waldeyer and their followers that it has been very widely accepted, and a plate showing the transition is shown in Dr. A. Martin's "Gynæcology." It is possible that greater importance has been attached to this condition of glands than has been warranted.

The question is of practical importance in regard to the microscopical diagnosis of suspicious affection of the cervix, for as it is admitted that the diagnosis cannot be made securely by the unaided eye nor by the touch, and as vaginal hysterectomy is now advocated, and, at any rate, free amputation of the portio vaginalis is indicated in all cases of undoubted cancer, even in an incipient stage, a great responsibility attaches to the microscopic examination.

In the first place, as Ruge and Veit expressly declare, in their majority of cases the carcinoma did not originate in the new-formed gland, but infiltrated the cervix as a "carcino-sarcoma," an aggregation of small cells lying in masses in alveoli of connective tissue. In such cases there was no evident connection with the epithelium of the surface with the glands.

In four out of twenty-two beginning cancers of the cervix, however, they found appearances of solidification of the glands and filling up with epithelium, which they describe and figure as a transitional stage in the development of the adjacent cancer. With much diffidence I venture to suggest that my studies of the changes in question have led me to different conclusions from these observers.

THE PLATES OF RUGE AND VEIT.

are not conclusive on this point.

Even when a whole series of glands lying adjacent to each other show occluded lumina on section, I cannot feel that the diagnosis of carcinoma is justified, but only that of adenoma. It may become destructive, but is not carcinomatous until changes occur in the connective tissue between the glands, when the boundaries of the glands are broken through by the growing cells.

Even when the new glands are thus involved manifestly in the carcinomatous growth, it has seemed to me they are invaded from without by the growth of cells in the surrounding tissue. . . . I have found no evidence that after filling up the lumen of a gland the proliferating columnar epithelium changes to the flat variety, and, breaking through the boundary of the gland, invades the surrounding tissue.

Moreover, in attributing so much importance to the fact that they found the lumina of some of the new glands occluded, Ruge and Veit have not noticed the explanation that precisely these solid acini or branches may be the first stage of their existence previous to the formation of the lumen.

Such a mode of growth is seen in the formation of new glands in the walls of a multilocular cystoma of the ovary. These little solid sprouts, lined with columnar epithelium, afterward become hollow and then dilate, forming cysts.

A similar mode of growth is seen in the female breast when rapidly enlarging, preparatory to the secretion of milk.

Where the microscope shows glandular degeneration, the surface bare of epithelium, the tissues heavily infiltrated with small cells, especially if the woman be fifty or over, we should not say that the microscope only shows chronic inflammation, but that

WHILE CANCER IS NOT PROVED, IT IS NOT EXCLUDED.

and should recommend a free removal or destruction of the suspected tissue.

Shall we, then, say that a case is not cancerous which shows no distinct structure of carcinoma on microscope section, only a glandular hypertrophy, with some of the glands filled with the epithelia and the stroma infiltrated with small cells, the surface denuded of its epithelial cells and irregular?

May we not reconcile the long contest between the two theories, which assign the origin of cancer respectively to the connective tissue and to the epithelial layer of the glands of the involved organ by supposing that the anatomical arrangement of cells, which clinically and microscopically we call cancer, is only the visible and outward sign of a morbific agent at present hidden from us?

The practical deductions which depend upon our speculative opinions as to the nature of cancer are of the greatest importance.

In the first place, if the disease comes from within, if it is a perverted growth of a part of the tissues, dependent on some original error of development, it is necessarily absurd to try to find, empirically, any medicine which should cure it.

If, however, it is an infection of some kind from without, we are justified in trying empirically, if as yet vainly, for some remedy which may overcome it.

Of more practical importance is the question of the utility of cauterizating the stump or cavity from which a cancer has been removed. There is considerable evidence which shows that surgical interference with a cancer is sometimes followed by a recrudescence of the disease more rapid and violent than the original disorder. If we consider that the operation opens veins and lymphatics which sometimes become infected with the morbific agent, we can better understand why a thorough cauterzation of the tissues left bare by the removal of a cancer of the cervix should be apparently so useful in lessening the chances of a return of the disease.

Dr. Franklin H. Martin, of Chicago, Illinois, read a paper with the title

A METHOD OF TREATMENT OF FIBROID TUMORS OF THE UTERUS BY STRONGER CURRENTS OF ELECTRICITY BASED UPON EXACT DOSAGE.

The author recognized in the treatment adopted by Dr. Apostoli a rational, harmless, comparatively painless and eminently successful mode of treating fibroid tumors of the uterus by electricity. It is upon these well-tried principles that Dr. Martin is able, after successful practical experiments, to lay down an exact line of dosage, enabling him to obtain all the beneficial effects of electricity without overstepping the limits of tolerance in the most susceptible or sensitive subjects.

The distinctively original feature of the paper was the description by Dr. Martin of his method of exact calculation of dosage, experiments being cited which showed that a certain local effect may be expected that an active electrode of a given surface from a definite strength of current passing for a certain length of time. The demonstrations proved that in order to get the characteristic local effects of electricity on the mucous membrane of the uterus, or to check hæmorrhage, upon a surface of one square centimetre, a current of twenty-five milliampéres passing for five minutes is necessary. One square centimetre is found to be about the surface represented in length of the uterine sound electrode. Upon this basis of calculation, the uterine canal which would require an Apostoli electrode twenty centimetres in length would require a current, if equal conduction took place from its entire surface, of five hundred milliampéres. The author argued that in many cases this strength of current would not be tolerated, and if it were there is no means of being certain that the sound conducts equally from so large a surface or that it comes in actual contact with the mucous membrane of the uterus in its entire extent.

In order to obviate this difficulty Dr. Martin has employed electrodes, concentrating the current to smaller portions of the uterine canal, so constructed that each portion of the canal may be treated by each succeeding operation.

HE RECOGNIZES BUT TWO VARIETIES OF OPERATIONS.

1. Positive inter-uterine galvanism, which corresponds to Apostoli's "positive galvano-caustique."

2. Negative inter-uterine galvanism, which corresponds to Apostoli's "galvano-caustique negative." These operations were fully described, from which procedures Dr. Martin believes we have a safe, painless, accurate, and rational method of treating fibroid tumors of the uterus. By this method all danger to the patient is avoided, such as may be observed in other treatments.

In conclusion, the principal advantages of this method were summarized under six headings: (1) It is entirely free from danger; (2) it is absolutely painless; (3) it invariably checks hemorrhage; (4) it rapidly reduces the size of tumors (5) it alleviates the neuralgic pain; (6) it is a system of treatment of fibroid tumors of the uterus based upon principles which make exact dosage possible.

Dr. Thomas Moore-Madden, of Dublin, Ireland, then read a paper entitled

SOME POINTS IN THE PATHOLOGY AND TREATMENT OF LACERATIONS OF THE CERVIX UTERI.

The pathology and treatment of lacerations of the neck of the uterus have received an amount of attention, in America and elsewhere, which would appear exaggerated were it measured by the comparatively scanty attention as yet accorded in Great Britain. When I read a paper on Emmet's operation at the closing meeting of the Dublin Obstetrical Society, the very name of that operation, or the circumstances calling for trachelorrhaphy had never been alluded to in the transactions of the association of British obstetricians and gynecologists.

In neither English nor American literature have I found sufficient recognition of the frequent complications, pure and immediate, arising from cervical lacerations in obstetric practice : viz., the causation of one of the most troublesome forms of post partum hæmorrhage ; and secondly, the occasional occurrence of puerperal septicæmia, as the direct result of lacerations of the cervix uteri. So far as he was aware, the advantages of amputation of the mutilated and hypertrophied cervix, in many cases of extensive stellate and bilateral lacerations, over trachelorrhaphy were not generally recognized.

THE RACIAL AND CLIMATIC CONDITIONS.
are not so obviuosly different in London and Dublin from the same in New York and Boston, and any method of treatment found useful on our side of the Atlantic, should, *cæteris paribus*, have a like effect in similar cases here. I therefore venture to submit my clinical experience to the judgment of the ninth International Medical Congress, and more especially my American brethren therein. It is to them that we who practice in the narrower limits, and perhaps more conservative atmosphere, of the old country are mainly indebted for our present acquaintance with this department of modern gynecology. I am convinced by my clinical experience, which is now tolerably large, that it is a far better and more rational practice, if any operative treatment be really required, to resort to the amputation of the entire extent of the mutilated and diseased cervix by either ecraseur or galvano-cautery. I need not say I do not advise this operation indiscriminately; indeed, I think the majority of cases of cervical laceration need no operative treatment specially, and such an operation as the removal of the cervix is not to be undertaken without due caution and, above all, real necessity. When thus justified, however, the amputation of the cervix, despite the protestations of some eminent gynecologists, is as unquestionably legitimate as any operation in gynecological surgery. By this procedure, when successful, not only may the surgeon rapidly and effectually remove every trace of a morbid condition, which, if uncured, would probably entail a life of continual uterine discomfort. We may also with certainty prevent the otherwise not improbable possibility of the lacerated and hypertrophied parts becoming the seat of malignant disease at a future period.

A paper by Dr. Leopold Meyer, of Copenhagen, Denmark, was then read on

CONTRIBUTIONS TO THE PATHOLOGY OF INFLAMMATION OF THE LINING MEMBRANE OF THE UTERUS (ENDOMETRITIS CORPORIS CHR.)

1. (*a*) In cases of chronic endometritis, as a rule, we find two varieties of cells, or, rather, of nuclei (as the limits of each single cell are often not distinct), in the inter-glandular tissue. One variety has smaller nuclei; these average as large, or a little larger, than red blood-corpuscles, are stained brightly by hæmatoxylin, carmine, Bismarck-brown, and the substance of the nucleus rarely presents a granular condition.

The second variety has a great resemblance to the so-called decidua cell.

(*b*) This second variety of cells is not only found in cases where the woman has been pregnant, but are seen in the most developed forms in women whose virginity is unquestionable.

(*c*) This second variety of cells seems, as the first variety, to be derived from the cells normally found in the inter-glandular tissue of the lining membrane of the uterus, the decidua. Cells of the second variety are found in the normal lining membrane during menstruation.

(*d*) Besides these two kinds of cells, we find regular connective-tissue cells and white blood-corpuscles in the inter-glandular tissue.

2. In cases of chronic endometritis, the epithelium covering the lining membrane of the uterus can preserve its character of a low columnar epithelium. but it frequently changes character altogether.

Dr. Alfred C. Garrett, of Boston, Massachusetts, read a paper on

TUMORS OF THE BREAST TREATED BY ELECTROLYSIS.

Many, or most of the tumors that so frequently occur in the human breast, we find can be completely cured, if treated while young, or new—that is, while in the first stage of existence, by certain mild applications of electricity.

In the first place, to obtain uniform success by this method, we must plan to find these tumors as soon as possible after they form in the breast, while they are in a curable stage in the majority of cases.

However, we already know we cannot assume that

EVERY MORBID LUMP

that grows or appears in the human breast begins from the first a simple or non-malignant tumor, though the most of them seem to do so, judging from the uniformly successful results of these treatments by electricity when applied to the selected, new, or recent tumor.

It is determined that here we must choose the form of electricity and method of application as carefully as we seek to find the curable cases; for we are not to resort to the usual electro-puncture needles, knife, wire, nor any active destructive electrolysis, nor any other means that shall produce solution of continuity. We are to employ simply surface applications of certain graduated, galvanic, steady currents, through peculiar, large, soft, and moist electrodes, so adjusted close to each side of the tumor as to cause this gently chemical current to completely permeate, and wash through the whole mass from side to side in its deeper parts, mainly in directing toward the axilla, for about a half-hour at each séance. It is not enough to simply apply the two electrodes to the surface of the breast or in any manner. Moreover, we need to use a milliampére metre to measure the current that actually passes through the tumor and gland; also a key-board that can enable the operator to pick up and increase the current, cell by cell, to the tolerant and efficient strength, which will be from ten to fifty milliampéres. The current required for each individual case cannot be stated in exact terms, as it is found in practice there is a wide difference in the resistance, tolerance, and effect in different persons; yet this point is of great importance.

The result is, that out of 186 tumors treated since 1864, a record of them having been kept and looked after, 157 disappeared and remained well. Several others did not quite obliterate, however, but left a small nodule, of the size of a chestnut, which in every case disappeared or remained benign.

---

AFTERNOON SESSION.

Dr. W. H. Weeks, of Portland, Maine, read a paper on

MYOMA IN PREGNANCY.

He considered the question of operation in these cases to be still under discussion. He introduced the paper by the relation of a case—a young lady, pregnant, having a large uterine fibroid which nearly filled the uterine cavity. He was in favor of allowing the pregnancy to go to term, and such was the opinion of a Boston gynecologist to whom he sent her for counsel. A New York gynecologist, however, advised immediate delivery by the induction of premature delivery. The method by which he would do this was to inject hot water and deliver under ether pervias naturales. If allowed to go to time laparotomy would be necessary. Two Boston gynæcologists agreed with him, and the author was obliged to surrender. He carried out the instructions of his colleagues to the letter. The patient died in less than an hour after delivery. She seems to have died of hæmorrhage and shock. When, as in this case, pregnancy is complicated by a large interstitial uterine fibroid, occupying and well nigh filling the cavity of the pelvis, is it better to induce abortion or premature labor or to allow gestation to go on to full term, and then deliver by abdominal section?

GUIDED BY THE LIGHT OF ABDOMINAL SURGERY,

I shall maintain that in the vast majority of cases, as stated in the above question, it is better to allow pregnancy to go to term, and then, if it is found that delivery is impossible *per vias naturales*, to resort to abdominal section without waiting till the patient's strength is exhausted by protracted labor. The operation best suited to such a case is Cæsarean section, Sänger's method, and then the removal of the ovaries, and, if possible, the fallopian tubes. Would it not have been better in this cause to have allowed the patient to go on to natural labor, and then performed laparotomy before she was broken down?

The author had sent out a letter of inquiry to prominent gynæcologists of this country and abroad. Nine out of thirteen physicians were in favor of the induction of premature labor. This proportion he hoped would be reversed.

Professor Graily Hewett, of London, England, opened the discussion and commended the paper very highly.

IT IS A THANKLESS DUTY

to bring an unfortunate case before the congress, and Dr. Weeks should have our admiration for doing so. I think that it is generally admitted that rapid evacuation of the uterine cavity in labor is a mistake. The operation in this case seems to have been done rather rapidly. Whether this had anything to do with the hæmorrhage and shock I do not know.

Dr. Trenholme, of Montreal, Canada, had had three cases of labor complicated with fibroids, which he related in brief. In one of these premature labor was brought on by a sessile tumor in the base of the uterus. The temptaton to remove the tumor was great, and he did it, but the patient died. In his second case he found a small fibroid, which he left alone. The patient did well for some time, but had a return of the trouble. He believed evacuation of the uterine contents should take place slowly.

Dr. Lawrence of Bristol, England, discussed the question.

SHOULD A WOMAN SUFFERING FROM FIBROID TUMOR BE ALLOWED TO MARRY?

This is a question frequently presented to the physician and gynæcologist. If the patient is not suffering from symptomatic troubles you may tell her that she will probably never be pregnant, but if pregnant she may probably have hæmorrhage and may die of it. With this understanding, if she persists let her marry. He reported eight cases in brief. In one of these, after a large number of pregnancies, the tumor disappeared entirely.

Dr. S. C. Gordon, of Maine, said that the case of Dr. Weeks was originally his own, and he felt like saying something. He agreed with Dr. Weeks that no abortion should be induced in this case. In his opinion the position of the uterus in this particular case forbade the operation. The position of the tumor influences very largely our procedure. He reported an illustrative case. In a second case which he cited he advised the induction of abortion, because the tumor involved the anterior wall of the uterus as low down as the os.

Dr. Alexander Dunlap, of Springfield, Ohio read a paper on

THE EARLY HISTORY OF OVARIOTOMY IN AMERICA.

This was a resumé of the trials and opposition with which the early American ovariotomists met in prosecuting their work. The author's own work, which is known to have been of great importance, though little of it has been published, was described minutely.

Early in 1843, with a very limited knowledge of medicine and surgery, he came in contact with a very peculiar case of ovarian dropsy which he had been led to consider as incurable. This he tapped several times, with the usual results. He then went into the history of his first ovariotomy with all the enthusiasm of forty years ago. After much delay the patient succeeded in forcing him to perform the operation, much against his will. He invited ten of his medical friends to witness the operation. They declined, saying that they could see enough people die without seeing them killed. One of them presented himself at the time. He was an old retired army surgeon. With this assistant and four students he operated, first giving the patient a teaspoonful of laudanum, after which he went to work. The operation was minutely described. The patient was placed in bed after the operation, with no especial shock, having watched every movement made with care and interest. She did well for four days, when she had a severe diarrhœa which was brought under control. She did well for a time, when she was taken with an excessive excretion of urine and died on the twentieth day after the operation. There was no septicæmia. He was convinced that she did not die from the operation but from the tappings, which had deranged the system and were probably the cause of the kidneys acting as they did. It is probable that had he operated when she first wanted him to do so she would have been alive to-day.

Dr. Kimball, of Lowell, Massachusetts, was then asked by the president to give his experience in early ovariotomy in the East. His story was quite similar to that of Dr. Dunlap, and his opposition as great. This opposition, he must say, came mostly from the New England metropolis, Boston. Being asked for his first case, he said his first case was one in which he did not operate. He then reported the first case in which he did operate about thirty-five or forty years ago. He invited ten physicians. During the operation he met with considerable difficulty in the form of nine cysts, and when he looked about for his assistants they had all left but one.

Professor N. Bozeman, of New York, N. Y., reviewed the revival of the history of ovariotomy after its abandonment by McDowell, of Kentucky, and its condemnation by the profession. Dr. Miller was the first to use chloroform as an anæsthetic in ovariotomy in America. It was in Terre Haute, Indiana, and Professor Bozeman gave the chloroform. He gave Dr. Dunlap much credit for his work, and especially for what he had done in recognition of the importance of adhesions.

Professor A. Cordes, of Geneva, Switzerland, read a paper on the

MEDICAL TOPICAL TREATMENT OF UTERINE CANCER.

The paper was divided into a general consideration of the subject: medical and surgical treatment, the opinions and treatment of Hippocrates and Celsus; the obscurity of the primary symptoms; the impossibility of hysterectomy in certain cases; adherence and extension to other organs; statistics; topical applications; the treatment of advanced periods of cancer by turpentine, the properties of this remedy, its application and preparation by M. Betrix, assistant to the clinique. The paper closed with a series of conclusions at which the author had arrived.

A paper was read by Dr. A. Lapthorn Smith, of Montreal, Canada, on

A NEW THEORY AND TREATMENT OF DISPLACEMENTS OF THE UTERUS BY ELECTRICITY, and one by Dr. Apostoli, of Paris, France, entitled

SOME NEW APPLICATIONS OF THE INDUCED OR FARADIC CURRENT TO GYNÆCOLOGY.

---

### SECTION ON OBSTETRICS.

### Third Day—Morning Session.

#### DISCUSSION ON CÆSAREAN SECTION.

Professor Alexander R. Simpson, of Edinburgh, Scotland, was in accord with the ideas expressed in Professor Lusk's paper, though he did not think the time had yet come when craniotomy could be entirely laid aside; its performance, however, should be restricted. In Edinburgh pelvic deformity was rare and craniotomy was rarely required.

The cases requiring abdominal section could be divided into two groups; first, those where the prognosis was rendered grave by pre-existing inflammatory conditions or sepsis, and, second, those favorable cases where we could choose the moment and place of operation, when we should nearly always succeed. At present he would be bold who would perform Cæsarean section by other than the Sänger method. Where the uterus was diseased and its removal would give the patient a chance, he would employ Porro's method.

Professor August Martin, of Berlin, Germany, considers the Sänger modification very important, and one which has rendered the operation safe. Since the adoption of this modification the section had been done in some cases where he thought version could have been safely performed. In moderate degrees of pelvic contraction we should ascertain whether delivery could not be effected in other ways, as by version, before performing the section. He had done the Cæsarean in pregnancy complicated with cervical myomata. Sänger had operated in similar cases. Where carcinomata of the organs in the pelvis endangered life we should endeavor to deliver the child and remove the growth at the same time. When the uterus was infected by septic material it should be removed. In a rachitic, kyphotic woman, with heart and lung-disease, the symptoms were so grave that he did not think the woman would stand normal parturition or the puerperal state; accordingly he had done abdominal section, saving both child and mother, but the latter finally died from her lung trouble. Much depended upon our knowledge of the technique; if we succeeded in doing the operation perfectly, either the Sänger or Porro operation was justified. The statistics of the Porro operation were rendered less favorable by Italian operations done on unfavorable subjects who were septic and in whom the operation was too long delayed. The Cæsarean section always gives us hope of perfect recovery, while the Porro operation prevents future maternity.

Abdominal section is indicated when it seems impossible to bring a living child through the pelvis (if care be taken not to operate too soon), when neoplasmata narrow the canal or endanger the progress of parturition; and when diseases are present in which the life of mother and child would be endangered by the process of parturition or the puerperium.

The Cæsarean section should be done when we have reason to believe that the patient can endure another pregnancy; the Porro operation, or total extirpation, when there is no hope of future maternity or where the disease, from its nature or seat, is probably fatal.

Dr. Joseph Taber Johnson, of Washington, D. C., reiterated the opinions expressed by Professors Lusk and Wathen. He believed that by delay and the attempt to perform other operations many lives were lost. He advocated the Porro operation when the uterus was septic or bruised. The wonderful results of foreign operators were due to their exceptional opportunities and skill, the operations being done by a few men, while here the operators were scattered, and many operations had been done in the backwoods.

Dr. Balls-Headley, of Melbourne, Australia advocated the Cæsarean section when the child, could not be born alive through the natural passages. There were also certain conditions, as carcinoma of the cervix, where other operative interference would allow the child to be born, but in these cases his experience taught him that the Cæsarean section offered a very much better prognosis. The operation was an easy one. In slight pelvic contraction the section gave good results. We should operate early, or, if the parts were bruised, substitute the Porro operation.

Dr. Doléris, of Paris, France, accorded with the views of Lusk and Martin. He believed in the use of the elastic ligature.

Professor W. W. Jaggard, of Chicago, Illinois, said, with reference to the *relative indication* for Cæsarean section, that in cases where the child could pass *per vias naturales*, when diminished in size, with safety to the mother, as, for example, in pelvic contractions of from 6 to 8 ctm. in the true conjugate, four considerations should receive attention :

I. Craniotomy does not require a higher degree of operative skill than every qualified obstetrician ought to possess, when proper instruments are employed, *e. g.*, Braun's curved trepan and cranioclast.

II. The mortality of craniotomy, when performed in time, and before exhaustion and infection of the woman, with adequate skill and antiseptic precaution, is, as remarked by Barnes, practically nil.

III. The consent of the woman, obtained without direct or indirect coercion, an essential condition of the relative indication, is seldom gained, if the facts be presented to her.

IV. That there is much sentimentalism with reference to the value of the life of the child in utero, as compared with the value of the life of the mother. This interest in the child is purely impersonal and scientific. The delight in saving the child's life is frequently that arising from the the success of a difficult scientific experiment.

Professor William T. Lusk, of New York, N. Y., said that the points made by Professor Jaggard were opposed to his recent investigation. With skill we could remove a living child where the contraction was only 7 to 10 ctm. Craniotomy was a dangerous operation, and, under three inches, required much skill and good instruments. He believed the Cæsarean section not more dangerous than the extraction of the child after craniotomy.

In his recent case the operation was done in the open ward, with the same preparations as for ordinary laparotomy. The children, according to his researches, did not die, as general opinion would have it. The Cæsarean section was easy to do. He thought there was danger, in the employment of the elastic ligature, from paralysis and inertia, caused by the compression. We did not want to encourage trying craniotomy and then Cæsarean section, but should make the latter the operation of election. Most of our cases had been done under circumstances which had rendered death inevitable.

### Afternoon Session.

Dr. J. A. Doléris, of Paris, France, presented a paper on the

#### TREATMENT AND SURGICAL RESTORATION OF THE CERVIX DURING PREGNANCY.

He first described a case where at a previous pregnancy the cervix had been very extensively lacerated. The patient was again several months pregnant, and suffered from a profuse, fetid, probably gonorrhœal discharge. There was severe vaginal pain, and the cicatrix of the cervical laceration was very painful when touched. Preliminary treatment did not relieve the patient and the cervix was sewed, four sutures being placed on either side; the vagina was filled with iodoform gauze; the result was excellent. There was no interference with the pregnancy.

This was only one of several similar cases where he had operated successfully.

A severe laceration accompanied by profuse and fetid discharge was very distressing, and might lead to abortion or puerperal fever.

He thought the danger of producing abortion by the operation was exaggerated, and that in the class of cases mentioned it produced good results.

Dr. Opie, of Baltimore, Maryland, recalled a single case where a severe bilateral laceration of the cervix had been operated upon; the woman proved to be pregnant, and aborted on the sixth day. There was objection to any operation about these parts during pregnancy, especially early pregnancy. Cervical operations were not without their special dangers at this time, and should be but rarely done.

Professor Leischman, of England, said that preconceived opinions often stood in the way of new truths. His preconceived opinion was that operations about the cervix were likely to produce abortion, especially when not done by the most skilled hands. Any operation during pregnancy, and this in particular, should be done only in rare and strictly defined cases.

Dr. Doléris responded that he had not advised the operation except in the well-defined cases he had mentioned, and in which he thought it would rather tend to prevent abortion.

Dr. Joseph Kücher, of New York, N. Y., read a paper on

#### ON THE RELATION OF THE ATMOSPHERE TO PUERPERAL FEVER.

He spoke of the importance of pure air in the lying-in room, and put the question: What influence has an impure air on puerperal fever? A *résumé* of the literature and statistics of the subject shows that the malaise of dissecting-rooms is more due to impure air than to absorption of septic material, and that true sepsis occurs only by inoculation through an open wound.

Overcrowding does not necessarily cause puerperal fever when septic infection is prevented. Pure air undoubtedly allows of more rapid convalescence; bad air depresses and allows the more easy access of septic infection; sepsis does not occur from bad air alone, but only from direct contact with septic matter.

Dr. Thomas More-Madden, of Dublin, Ireland, presented a paper

ON THE PREVENTION AND TREATMENT OF PUERPERAL SEPTICÆMIA, which was read by the secretary.

The author considers all forms of septic fever consequent on parturition, and occurring within the puerperal period, as various manifestations of a specific puerperal fever. Puerperal sepsis may originate in three ways, viz., from inoculation with the micrococci of clinically allied diseases, such as erysipelas or scarlatina; from infection by the pathognomonic, chain-like micro-organisms evolved by other puerperal fever patients; or the disease may arise from auto-infection with self-generated septic matter. Although absolute immunity from puerperal fever must be considered hopeless, its prevalence may be much diminished, and its virulence minimized, by the rigid observance of certain precautionary antiseptic and hygienic measures.

For prophylaxis the author strongly recommends the administration, during the latter months of pregnancy, of the chlorates of iron, potash and quinine. Strict attention to the patient's local and general hygienic and aseptic condition is insisted upon. The author uses a carbolized intra-uterine douche daily throughout the puerperium, together with large doses of ergot for the same time.

In the treatment of puerperal fever he relies primarily on the maintenance of the patient's strength by suitable nourishment and stimulants; secondly, on the daily washing out of the uterine cavity with hot water, plain or medicated; thirdly, on full doses of quinine and turpentine —which latter drug he believes to be especially valuable in every form of puerperal fever.

Professor Chas. Warrington Earle, of Chicago, Illinois, presented

A STUDY OF CERTAIN QUESTIONS IN CONNECTION WITH PUERPERAL FEVER, WITH PARTICULAR REFERENCE TO THE USE OF INTRA-UTERINE DOUCHE AND CURETTE.

After a discussion of various theories, he concluded that puerperal fever was in every instance produced by infection from without. There was no such thing as autogenetic infection. No one could now disbelieve the germ-theory. Experience had shown that it was impossible for decomposition to occur without the presence of bacteria. There were many ways and many forms of infection. In a larger number of cases than we could suspect, debris, bits of placenta, decidua, etc., were left in the uterine cavity, which were, in our present modes of practice, infected by patient, nurse or doctor.

The only rational treatment was to remove all decomposing material, and prevent a local poison from invading the entire system, and producing general sepsis.

Any marked rise of temperature in the puerperal woman should be investigated, and, if not plainly due to other causes, the genital tract should be suspected. A vaginal and uterine douche not reducing it, the uterus should be curetted, using a large, blunt instrument with all antiseptic precautions and great gentleness; the curetting to be followed by an intra-uterine douche to carry away all loosened shreds.

The author cited cases where the curetting had been done with the most gratifying results, and in no case did he know of any harm resulting from it. He did not wish to be understood as advocating the indiscriminate use of this measure, it being indicated only in those cases where the high temperature persisted after an intra-uterine douche, when it was extremely valuable.

Dr. R. Lowrey Sibbet, of Carlisle, Pennsylvania, read a paper on

THE PREVENTION OF PUERPERAL FEVER.

The contagium was always from without, and was never autogenetic. It was carried always by the medical attendant or nurse. Aseptic cleanliness on their part was the best prophylaxis. He had always kept a disinfectant on his wash-stand, and had never lost a case of puerperal fever. He did not believe in the intra-uterine douche in private practice; it was dangerous, and could not be trusted to an unskilled nurse.

Dr. Doléris, of Paris, France, thought that in practice we were ahead of theory. He used the douche and the curette, or the milder intra-uterine brush, in cases such as Professor Earle had indicated. He had used these measures with the very best success in many cases, from the second to as late as the seventeenth day.

Professor W. W. Jaggard, of Chicago, Illinois, would treat the uterus when mild infection had already begun. He vigorously protested against Madden's use of the douche, daily, and was more in accord with the Vienna school, where the uterus was washed out thoroughly with a weak (two and one-half per centum) carbolized solution, and a bacillus of iodoform weighing from sixty to seventy grains introduced. No more interference. The use of the douche not repeated, unless in exceptional cases. There was no danger from iodoform poisoning.

Dr. J. F. Y. Paine, of Galveston, Texas, in twenty-four years had but one case of puerperal fever. The best prophylaxis was to secure firm contraction of the uterus and to remove clots. The uterine douche was meddlesome and hurtful, in many cases. Cleanliness, rest, quiet, good air, and good diet were all important. Quinine and iron might be given in some cases.

Dr. D. T. Nelson, of Chicago, Illinois, mentioned a very grave case of puerperal septicæmia where sulphuretted hydrogen gas was used, as in Bergeon's method in phthisis. At first the sympoms became more favorable, then diarrhœa set in. The treatment was contined cautiously, but rectitis and hæmorrhage appeared, and the patient died. Here, at first, the treatment seemed to be beneficial, but later, no doubt, hastened her death.

In a second case, in the third week of typhoid fever, a miscarriage occurred at the third month. The fever became septic, with a characteristic scarlatinal rash. The same treatment was used, employing the gas in smaller quantity, and with immediate amelioration and recovery.

Dr. William T. Stewart, of Philadelphia, Pennsylvania, indorsed Professor Hewitt's views, and thought the liquor sodæ chlorinata, ℥ ij. to O ss., the best for vaginal douching.

Dr. Lloyd Roberts, of Manchester, England, used quinine, irrigation, and the curette when necessary. Mild cases would get well without treatment. He did not agree with Dr. Sibbet that the doctors and nurses so often carried the disease. He considered the mercuric-bichloride douche dangerous when strong; calomel was used successfully forty years ago. Could it not have had an antiseptic action?

Dr. Cameron, of Montreal, Canada, did not think we could treat the disease intelligently until we realized that puerperal fever and puerperal septicæmia were synonymous. There was no such thing as auto-infection; endosepsis was a pernicious myth. His views were in accordance with those of Professor Jaggard. Antiseptic precautions were to kill or to prevent the entrance of germs. There was a very general lack of knowledge as to when or how to use antiseptic measures. The douche and curette were valuable in the right place, but harmful in others.

When one has used the douche, iodoform, and the antiseptic pad, why use other local measures? The canal had been sterilized. Food, stimulants, rest, and iron were important. There was no specific treatment. Each case should be studied. Local conditions required local treatment, general states general treatment.

Professor Charles Warrington Earle, of Chicago, Illinois, thought the ground had been very thoroughly gone over by Dr. Cameron. He believed in adopting absolute antiseptic precautions beforehand. If possible, there should be clean, intelligent nurses. The cause of the sepsis should be sought; if in the uterus, one should clear it out and the patient would probably recover.

Dr. Rodney Glisan, of Portland, Oregon, presented a paper on

### CONSERVATIVE OBSTETRICS; WITH SPECIAL REFERENCE TO THE REMOVAL OF THE SECUNDINES AFTER ABORTION, AND TO THE TREATMENT OF THE THIRD STAGE OF LABOR.

He thought that the expectant method of treating retained secundines after abortion and the placenta after labor was unsafe in private practice, especially when the physician resided at a distance from his patient; yet it might succeed fairly well in hospitals under the constant vigilance of experienced practitioners.

He approves of the immediate removal of the secundines after abortion in all cases where the cervix is somewhat dilated or dilatable, as is generly the case for an hour or so after the expulsion of the embryo, and in all cases of septicæmia or dangerous hæmorrhage, no matter when they occur. When neither of these accidents is present and the cervix is closed, he does not advocate the immediate and forcible removal of the secundines, but would wait a more favorable condition, when the finger could be easily inserted, moderate hæmorrhage being controlled by ergot, the tampon, etc. No instrument in these cases was so safe, trustworthy, and generally useful as the finger. He adopts the bi-manual method, depressing the uterus with one hand to within reach of the finger of the other, giving an anæsthetic, if necessary.

In the removal of the placenta, during labor, the author used the Credé method, supplemented by moderate traction on the cord. He does not believe that moderate traction on the cord, when the uterus is well contracted and properly grasped by one hand externally, is attended by the least risk of inversion or of increasing the hæmorrhage by a suction-like process of the placenta on the cavity of the womb. He thinks that traction upon the umbilical cord as an aid to delivery of the placenta ought not to be abandoned.

Professor Graily Hewitt accorded perfectly with Dr. Glisan. In cases of abortion there was often great difficulty in passing the internal os. We must not expect the os to be open until the abortion had continued for some time. Instruments other than the finger were dangerous. He was much impressed with the importance of not allowing the secundines to remain long in the uterus.

Others present expressed the same views.

---

AFTERNOON SESSION.

Dr. Edward Henry Trenholme, of Montreal, Canada, presented a consideration of

INTERNAL UTERINE HÆMORRHAGE, THE RESULT OF OVER DISTENSION OF THE UTERUS FROM HYDRAMNIOS.

The author pointed out some of the causes of hydramnios and the serious result of such distension. The distension caused deficient nutrition of the decidua which allowed it to rupture, causing hæmorrhage from the site of the tear. The blood might clot *in situ* or pass between the layers of the deciduæ. The hæmorrhage began with severe pain and sense of fullness, signs of internal hæmorrhage. We should forestall the danger by causing premature labor before the hæmorrhage occurred, that is, as soon as the distension becomes excessive. Should bleeding have occurred, it is necessary to wait until the vessels have closed before one attempts treatment.

A posthumous paper by Dr. W. T. Taylor, of Philadelphia, Pennsylvania, on

MATERNAL IMPRESSIONS AFFECTING THE FŒTUS.

was read by title.

---

### SECTION ON GENERAL SURGERY.

THIRD DAY—MORNING SESSION.

Dr. W. N. Hingston, of Montreal, Canada, discussed Professor McLean's paper of the preceding day. He remarked that he agreed with him in all but one thing, viz., regarding abdominal section. He had operated in three cases ; in two of these cases he had made the abdominal section, and both patients died from shock. In the second case he performed the lumbar incision and the patient survived, and there was but little disturbance of the system after the operation. The speaker considered the lumbar incision advisable in all cases where possible.

Mr. Edmund Owens, of London, England, thought there were three factors which should guide us in the operation.

If there was a doubt about the operation, it should be done through the abdominal wall. Again, the size of the tumor must be considered. And in what line of the profession is the operator called, gynæcology or surgery? This latter will often decide the point according to the ground he is apt to go over.

Dr. Hardy, of Indianapolis, Indiana, mentioned a fatal case where the median incision was made.

Dr. F. Lange, of New York, N. Y., used the lumbar incision ; and if the tumor was too large to be removed, he thought it a good plan to incise it and perform a second operation when it had been reduced.

Professor McLean, in conclusion, remarked that perhaps he was not quite understood, as he had distinctly stated in his paper that where the lumbar incision could be performed it was preferable. In the cases he mentioned he had no choice. His first case the speaker thought was an ovarian tumor, and naturally made section in the median line.

Dr. Richardson, of Boston, Massachusetts, read a paper on

GASTROTOMY FOR FOREIGN BODIES IN THE THROAT.

A patient (male), thirty-seven years of age, was presented to the section, from whom the doctor had removed a plate with four teeth attached from the lower portion of the æsophagus, about one and one-half inches from the stomach entrance, by performing gastrotomy. When first seen by the doctor, he could discover no obstruction, but the man was, however, retained in the hospital. He was finally discharged apparently well, the pain at the point indicated by him having subsided, and he was able to eat his meals. Eleven months later he returned very much emaciated, the pain had returned and was much more severe ; gastrotomy was decided upon and the operation performed, the opening being made large enough to admit the hand ; the plate was then discovered in the location stated. Owing to its long retention a small abscess had formed there, which subsequently ruptured, but the patient made a rapid recovery.

The speaker remarked that had he been certain that the plate was there, he was positive he could have removed it with the forceps ; but the difficulty lies in locating, and also the location of the cricoid cartilage and the cardiac opening. He had operated in sixty cases, and found the average distance from the incisors to the cardiac opening to be fourteen and one-half inches ; the longest was seventeen inches, and the shortest ten and one-half inches.

The writer described minutely the anatomy of the parts and location of the plate removed ; showing that by gastrotomy or æsophagotomy the æsophagus could be reached at all points with ease.

Professor F. S. Dennis, of New York, N. Y., presented some specimens of *scarlet fever bacilli* sent by Professor Lee, of Edinburgh, Scotland. He then read a paper on

AMPUTATION OF THE HIP-JOINT FOR SARCOMA.

The case was quoted in order to induce surgeons to report such cases and the results. Out of twenty-eight cases of sarcoma of the thigh, only two were living.

The case at present was a young man seventeen years of age, whose family history was excellent. Five months before entering the hospital the swelling in the left thigh commenced; it had been aspirated, and ten ounces of fluid removed. The tumor when seen was ten inches in length, and twenty-seven in circumference, the inguinial glands were slightly enlarged.

The diagnosis of sarcoma was made, and amputation at the hip-joint was decided upon. No elastic bandage was applied to the leg, but a strong rubber band was passed around the thigh above the joint, which controlled all hæmorrhage, and the operation was a bloodless one. On the sixth day the dressings were removed, and the wound was found to have united by first intention; temperature did not rise over 100° F., and only reached that point on one day.

The points worth noting are: (1) this patient had a sarcoma, with no hereditary taint; (2) there was no exciting cause; (3) the great rapidity of the growth; (4) absence of metastasis; (5) rapid return to health, as the boy is perfectly well now: (6) importance of secondary hæmorrhage; (7) unfavorable prognosis in his condition; (8) the diagnosis was confirmed by the microscope in the hands of Dr. Grower; (9) abscess limited to shaft of the bone; (10) absence of a spontaneous fracture of the femur; (11) large size of the mixed cells of the sarcoma; (12) radical removal of the tumor by amputation at the hip-joint, not at the upper third of the thigh.

Dr. Garmody, of New York, N. Y., read a paper on THE SURGICAL TREATMENT OF TRAUMATIC INSANITY BY MEANS OF THE TREPHINE.

The speaker reported the case of a young woman who was struck on the head with a brick, causing depression of the skull; she had been trephined shortly after, but ten years later she began to exhibit symptoms of insanity; trephining was suggested by the speaker, and the operation followed out, the space, after the operation, being three and a half inches by two inches. No elevation of temperature followed, and at the end of twenty-one days she was perfectly rational; the wound united by first intention.

Sir James Grant, of Canada, remarked that the case was an interesting one, and in his experience of brain surgery he had found that the lower type of brain would bear a fracture of the skull much better than those of an intellectual type. He quoted the case of a mill-hand who fell out of a window and fractured his skull. The speaker was sent for, and on his arrival found the man walking about, and yet there was a depression in the back of the skull in which the finger could be inserted; the brain, he considered, became very sensitive in educated individuals,

---

### SECTION ON MILITARY AND NAVAL SURGERY AND MEDICINE.

THIRD DAY—MORNING SESSION.

The first paper of the day, upon

THE PROPER TREATMENT OF PENETRAING WOUNDS OF THE JOINTS, was read by the author, Professor Frederic Hyde, of Syracuse, New York.

After a critical review of the history of the subject, of the methods advocated by various civil and military surgeons for the treatment of these wounds, and the textural changes resulting therefrom, the author stated that the result of the wound, whether incised, lacerated, contused, or punctured, depends upon the intensity of the inflammation and the destruction of tissue, and presented his views as to the treatment. These may be summarized into: The arrest of hæmorrhage; treatment of shock; the removal of foreign bodies from the wound, which should be enlarged, if necessary. In all cases of suppuration a direct opening should be made for its evacuation. After any form of injury an effort should be made to save the limb; perfect immobility should be secured by plaster of paris or other fixative dressing; finally, pain should be alleviated, and the general condition be improved by stimulants, good food, and the like, according to indications. The treatment of the injury throughout should be antiseptic. The speaker contrasted the earlier treatment of joint-wounds, which forbade the opening of a suppurating synovial sac, with the treatment of to-day, and characterized the former as the spoliative and the latter as the saving treatment. Amputation should only be resorted to as a *dernier ressort*.

The next paper, upon the same subject, by Dr. George L. Porter, of Bridgeport, Connecticut, was then read. It had especial reference to the injuries of the larger joints, particularly that of the knee. The treatment should be adapted to each individual injury; aseptic precautions, complete rest, the ice-pack, and expectant treatment generally the author thought to be applicable to most cases.

Dr. Eli A. Wood, of Pittsburg, Pennsylvania, read a short paper on this important subject, which in the main coincided with the views as expressed by Dr. Hyde.

Dr. Marston, of England, favored amputation.

Dr. Janes, of Waterbury, Vermont, stated that his experience had been an unfortunate one, all his cases having died.

Professor E. H. Gregory, of St. Louis, Missouri, inveighed against resorting to amputation, and favored the opening of the wound under aseptic precautions and removal of foreign bodies, and careful supporting treatment.

Dr. G. T. Langridge, of England, spoke of his large experience in wounds of the knee-joint produced by machinery; under aseptic treatment a very large proportion of cases recovered completely.

Dr. L. von Farkus, of Buda-Pesth, Hungary, stated that during the late Servo-Bulgarian war there were observed nineteen cases of gun-shot penetrating wounds of the knee-joint, all of which were, with one exception, treated by fixation by the plaster of Paris bandage, after free opening under antiseptic precautions and drainage; fever was absent in all cases, and all recovered, including the exception mentioned, which was a case of injury so serious as to require amputation.

---

### Third Day—Afternoon Session.

The paper of Dr. W. S. Tremaine, U. S. army, entitled

IS LAPAROTOMY FOR GUN-SHOT WOUNDS OF THE INTESTINES FEASIBLE IN MILITARY PRACTICE AND ITS TECHNIQUE?

and another upon

THE TREATMENT OF PENETRATING WOUNDS OF THE ABDOMEN, WITH WOUND OF THE INTESTINES, by Dr. Frances Patrick Staples, of the British army, were read by title.

An abstract of a paper upon.

THE PROPER TREATMENT OF PENETRATING WOUNDS OF THE ABDOMEN, by Dr. S. T. Armstrong, U. S. Marine Hospital Service, was, owing to the absence of the author, read by Dr. Smith, of the army. The main points made by the writer were, that the treatment must depend upon the part injured, and that rest, sedatives, expectant treatment, with laparotomy as a last resort should generally characterize it.

The next paper, also upon this important subject, entitled

ON NON-FATAL PENETRATING GUN-SHOT WOUNDS OF THE ABDOMEN TREATED WITHOUT LAPAROTOMY, by Henry Janes, M.D., of Waterbury, Vermont, was read. The author maintained that the tendency at present is to too much operating upon the abdominal cavity. He had tabulated the cases observed by himself during the late war, of penetrating gun-shot wounds of the abdomen, covering twenty-seven cases, eighteen of which were complicated by lesions of the abdominal viscera, and other cases of injury to pelvic and abdominal organs. In none of those cases had operations been performed, except for the purpose of removing fragments of bone, the missiles, or other foreign bodies.

The papers upon the subject of Penetrating Gunshot Wounds of the Abdomen, elicited an interesting discussion, in which Dr. Thomas G. Morton, of Philadelphia, Pennsylvania, participated.

The first point to be considered, he said, in the treatment of these wounds, is whether the patient is in a proper condition for an operation; upon this depends the success of many operations. Another point of great importance is the positive knowledge of the existence of a perforation of the intestines or other abdominal organs, or even of a mere penetration of the peritoneum, without such complication. There being no doubt in the surgeon's mind as to these points, he should not have the least hesitation in opening the abdomen. There exists, in all cases of penetration, a possibility of loss of blood from some severed vessel, and of the escape of fæcal matter into the peritoneal cavity.

The abdomen should always be opened under strict antisepsis; thus the operation is attended with little danger. In cases of stab-wounds of the abdomen, with perforation of the peritoneum, he would advise making an incision sufficiently large to enable the surgeon to clearly see what he is doing, and, having secured any bleeding vessels, washing out the cavity with a warm solution of bichloride of mercury, a 1–10,000, until the water returns perfectly clear, and then sewing the wound in the usual way. He prefers, in the majority of cases, to make the incision over the middle line, except in perityphlitic abscess, when he makes a curvilinear incision over the most prominent parts of the abscess. His experience has taught that a large incision is better than a small one, and safer in every respect. For a gunshot wound he proceeds in the same manner. In a boy seventeen years of age, with a penetration of the transverse colon, and a young negro with a similar injury, upon whom he lately performed this operation, the temperature did not exceed 101° and 102° F., respectively, and this for the first day only, recovery being perfect and rapid in both cases.

Dr. Henry Janes, of Waterbury, Vermont, asked Dr. Morton how long a time such an operation as he had described usually required.

Dr. Morton, in answer, replied that the time varied somewhat, according to the seat and extent of the injury, but that from one hour and a half to two hours was, as a rule, sufficient.

Dr. Varian, of Titusville, Pennsylvania, desired to know Dr. Morton's opinion as to the practicability of performing this operation upon the battle-field.

Dr. Morton, in reply, thought it would be difficult, but that the numerous objections which had been given concerning the impracticability of operating were easily disposed of. In the first place, the probable abundance of pure spring water in the neighborhood with which to prepare the solution of the bichloride—which can easily be carried in the pocket—and the removal of the patient to some field hospital, were, of themselves, conditions which would enhance, rather than diminish, the chances of success. He considers it the surgeon's duty, in the light of recent experience and observation, not to temporize, but to make an effort to save the patient; this operation can do it, while the expectant treatment involves many dangers. He is confident that something can be devised for use on the battle-field; a modified antisepsis is better than no antisepsis at all.

Dr. Daniel Smith Lamb, of Washington, D. C., asked how large a wound would induce him to operate.

Dr. Morton replied that the slightest perforation of the peritoneum was an indication for the operation, for it is well known that these small wounds may be, and are often, followed by grave results. He said that he advocates the opening of the abdomen in typhoid fever when perforation of the intestine by ulceration has taken place; such an operation gives the patient the greater chance of recovery.

The President, Professor Smith, adverted to the great importance of this subject. What we want, to deduce conclusions for our future guidance, is an accumulation of facts. Dr. Morton, by his remarkable success in these cases, which success was not, it may be confidently asserted, the result of mere chance, and the New York surgeons, with their recent experience, had, he thought, been accumulating such facts very rapidly. In the light of these facts the legal responsibilities of the surgeon should be considered. The President concurred with Dr. Morton in his views as to the possibility of modifying antisepsis to meet the exigencies of the battle-field.

Dr. Moore, of Richmond, Virginia, read a paper entitled

THE TREATMENT OF PENETRATING GUNSHOT WOUNDS OF THE ABDOMEN.

The author gave statistics of cases of this nature and the rate of mortality, and quoted at length from reports of the French and British armies.

No surgeon had, in his opinion, had sufficient experience with this operation, which was comparatively a new one, to be considered an authority on disputed points. He spoke of the difficulties of diagnosis of perforation, and advocated the exploration of the wound with some delicate instrument. The incision should be made only sufficiently large to allow a thorough exploration, and the earlier after the reception of injury, barring the occurrence of shock, the greater were the chances for recovery. It is the opinion of Sir William Malcolm that if the operation be delayed twenty-four hours no case ever recovers.

In conclusion, he advocated supporting measures and the use of quinine hypodermically, previous to the operation, to diminish the liability of resultant shock.

Dr. Varian's views were in harmony with those of Dr. Moore, so far as civil practice is concerned, but the question still to be discussed was the treatment of such cases upon the field of battle.

Dr. Watson, of Jersey City, New Jersey, agreed with Dr. Morton.

Dr. Bentley, of Little Rock, Arkansas, dissented from the opinion of Dr. Morton, and regarded the application of the method of treatment as impossible on the battle-field, as it is an operation which requires skill, time, and care. He, however, recognized its value in private and hospital practice, where all the conditions are favorable.

---

### SECTION ON PATHOLOGY.

### Third Day—Morning Session.

THE PIGMENTATION OF THE SKIN AT THE ARTICULATION OF THE PHALANGES IN CHLOROSIS.

Dr. Pouget, of Cannes, France, read a paper on this subject, in which he directed special attention to pigmentation of the knuckles, a fact first noticed by Bouchard, of France.

Dr. Thomas Taylor, of Washington, D. C., read a paper on the

CRYSTALLOGRAPHY OF FATS, in which he directed special attention to their condition and relation to health and disease.

THE ETIOLOGY OF LIVER ABSCESS

was the title of a paper sent by Dr. Kartulis, of Alexandria, Egypt, and read by Dr. J. S. Grant (Bey), of Cairo.

Professor Charles W. Earle, of Chicago, described

FIBROID DEGENERATION OF THE PANCREAS, of which he had reported cases, and which was liable to be mistaken for cancer.

---

AFTERNOON SESSION.

Dr. John North, of Keokuk, Iowa, read a paper on

THE PATHOLOGICAL RELATIONS OF PTOMAINES AND LEUCOMAINES.

The paper was discussed by Dr. Johnson and Dr. Keller, and was followed by a paper on

TYROTOXYCON, by Professor Victor C. Vaughan, of Ann Arbor, Michigan.

---

### SECTION ON DISEASES OF CHILDREN.

THIRD DAY—MORNING SESSION.

Dr. Cyrus Edson, of New York, N. Y., read a paper on

THE MILK-SUPPLY OF CITIES, calling attention to the great importance of improving and maintaining the standard of milk-supply by banishing adulterated and infected milk. He advocated the examination by veterinary inspectors, from time to time, of every herd of cattle in the state, in order to secure the destruction of all animals suffering from tuberculosis, and the quarantine of those liable to cause contagious disease.

Professor Albert Leeds, Hoboken, New Jersey, stated that the work done by inspectors in preventing the dilution of milk is important from a commercial rather than a sanitary point of view, as water is in itself harmless. The best work is done when official inspection is extended so as to include the pasture, the stall, and the dairy. Skilled and scientific effort in this direction would prevent the spread of infectious diseases from cattle to man.

A paper was then read by Dr. Paul Redard, of Paris, France, on

THE TREATMENT OF ERECTILE TUMORS BY ELECTROLYSIS.

The writer described in detail the method proposed by Ciniselli de Crémorne, which, with some modifications, he had found satisfactory in a large number of cases in dispensary practice. The operation is moderately painful, and the effect rapid, with no suppuration or scars.

Dr. De Valcourt, of Paris, France, then read a paper on

THE TREATMENT OF TUBERCULOSIS BY SEA-BATHING.

---

AFTERNOON SESSION.

Dr. Isaac N. Love, of St. Louis, Missouri, opened the session by reading a paper on

INFANTILE MARASMUS.

He reviewed a class of cases distinct from those caused by tuberculosis, syphilis, and intestinal catarrh. In an advanced stage they present a picture the principle features of which are wasted muscles, prominent bones, a loose and dry skin, and face withered, wrinkled, and wan. Careful study of a large number of such cases had led him to the following conclusions:

1. Infantile marasmus is dependent primarily on torpidity and inactivity of the glandular system, and is aggravated by unsuitable, overabundant, or insufficient food and unsanitary surroundings.

2. It is of the first importance, in treatment, to arouse secretion and excretion, the best remedy being calomel in doses of one-twelfth of a grain, with the free administration of water; both of these agents exciting glandular action, stimulating the secretion of the digestive juices, and promoting diuresis and intestinal secretion.

2. "In the matter of diet, the mother's milk is the best, and some other mother's milk the next best."

4. In extreme cases it is best to administer soluble foods in the form of baths, and to practice gentle friction and massage, with an occasional bath in water containing a diffusible stimulant.

A paper was then read from Dr. William Henry Day, of London, England, entitled

SOME OBSERVATIONS ON HEADACHES IN CHILDREN, AND THEIR RELATION TO MENTAL TRAINING.

The writer considered the characteristics of nervous, frontal, and neuralgic headache, and the condition known as irritable brain. One form or the other is the first and most persistent symptom of the breaking down which follows the mental over-pressure which now prevails in education, and in the competitive examinations for entrance in every profession and in every branch of the public service. The irritable brain is not infrequently accompanied by myopia, hypermetropia and astigmatism.

Dr. William S. Dennett, of New York, N. Y., recognized the type of headache to which Dr. Day had referred as associated with refractive troubles, as well as the occipito-cervical discomfort associated with muscular insufficiency. It would be well if all cases of incipient myopia were attended with a forewarning headache.

Dr. William D. Booker, of Baltimore, Maryland, then read a paper, entitled

A STUDY OF SOME OF THE BACTERIA FOUND IN THE DEJECTA OF INFANTS AFFECTED WITH SUMMER DIARRHŒA.

Twelve different varieties of bacteria had been isolated. Eleven were bacilli and one belonged to the cocci. Two of the bacilli only liquefy gelatine. Their action on milk is as follows: Some coagulated casein with acid reaction and evolution of gas, one variety caused coagulation with akaline reaction, one gave the milk a peptonized appearance, other varieties caused no perceptible change.

In guinea pigs, young kittens, and white rats and mice the post-mortem results were fully detailed in the paper, and an interesting discussion followed.

---

### SECTION ON LARYNGOLOGY.

### Third Day—Morning Session.

Dr. H. H. Curtis, of New York, N. Y., being absent the paper by him on

SURGERY OF THE NASAL SEPTUM AND TURBINATED BODIES.

was read for him. He classified various deformities of the septum so as to more accurately describe the different operations. Operations on the turbinated tissues were disposed of in systematic manner, and attention was given to catarrhal conditions of the ethmoidal cells.

Dr. F. Massei, of Naples, Italy, sent a paper on

PRIMARY ERYSIPELAS OF THE LARYNX, which, in his absence, was read for him. It opened by quoting a list of authors who admitted that there was a primary erysipelas of the larynx. It is fortunately a very rare disease, and no good clinical history is given showing the laryngeal appearance. The disease is usually secondary to erysipelas elsewhere on the body; where primary it is found in hospitals where the air is bad, and where the patient's bed is near that of another who has an attack of erysipelas. The disease may begin in the throat and attack the skin later. In 1885 his attention was called to cases in private families where there was primary erysipelas of the larynx. The cases had not been exposed, to his knowledge, to infection from other cases of erysipelas. There was no manifestation of the disease elsewhere on the body. He noticed one form where the local symptoms were most prominent, and another where there was early collapse. It may extend to the lungs without any external appearance of the disease.

Early symptoms are, difficulty of swallowing and talking, change of voice, with pain; fever is high, and pulse rapid. Epiglottis swollen, mucous membrane of larynx intensely congested and reddened, also much swollen. Narrowing of glottis even to suffocation may occur as symptoms increase in severity, and we find aphonia and dyspnœa, caused by swellings and and œdema over arytenoid cartilages and ary-epiglottic folds. The swelling has a way of migrating from one part to another, may sink down on one side and the other become rapidly tumefied. The swelling of a part is preceded by intense congestion. Frequently there is an eruption of blisters or bullæ. These burst and form crusts with mucous and blood. Fever is more or less relapsing, temperature varies from 40° to 38° C., but is not periodical in its changes. Lymphatics are very sensitive, and neighboring glands are swollen and inflamed. After several relapses of temperature, we may have resolution and recovery, or death from asphyxia if tracheotomy is not resorted to, or collapse from extension to the lungs. More apt to extend to the lungs in the aged. In treatment use ice or coil to the larynx, also counter-irritants; use bichloride mercury, one to two-thousand, as a spray. If patient cannot swallow food through a tube when œdema or bullæ interfere seriously with breathing, then scarify, and resort to tracheotomy if necessary. In migrating from one part to another, it may be rapidly fatal so as to give no time for action. The treatment must be expectant and should vary with the symptoms.

Professor F. O. Stockton, of Chicago, Illinois, saw a case of a powerful and healthy man, who came to Chicago for nasal trouble. He contracted what he thought was severe cold, and had symptoms rapidly increasing in severity; pulse, 140; temperature, 103½°, preceded by severe rigors. There was great difficulty in breathing, with aphonia. The epiglottis was thick and red, so also were the arytenoid and folds between, and thickly covered with small blebs. There was more or less œdema over the arytenoids, with numerous blisters. The Ventricular bands covered the true vocal cords, and almost completely closed the glottis. He tried intubation, which worked perfectly, to relieve dyspnœa; but the redness of the erysipelas extended over the pharynx to post-nasal space and through the nose, then over face and eyes to the forehead. The patient recovered.

Dr. Benham, of Pittsburgh, Pennsylvania, thought it more common than is usually supposed, and there is no disease more distressing or more likely to tax the physician's resources much more. He has never seen a primary case of it. Instant relief sometimes results from scarification. Hè cannot understand the sudden recovery that sometimes occurs.

TWENTY YEARS OF LARYNGOLOGICAL WORK IN THE CITY OF MEXICO

was the title of a paper sent by F. Semeleder, M. D., of Mexico. It treated chiefly of the difficulty of getting books and instruments early in his career, and described how long laryngology was unknown there. He gave a list of prevalent troubles, and the various operations which he had performed.

PRESENT STATUS OF THE GALVANO-CAUTERY IN THE TREATMENT OF THE DISEASES OF THE UPPER AIR-PASSAGES, ILLUSTRATED BY INSTRUMENTS AND THE DESCRIPTION OF CASES, was the title of a paper by Professor F. B. Eaton, Portland, Oregon.

The author being absent, the paper was read and the instruments exhibited.

Inventive faculties are stimulated continually to devise more radical methods of treating nasal catarrh. It is best to avoid over-medication, overtreatment. He considers specialists a necessary evil. There will be a reaction and less operative interference result. He defends the galvano-cautery against charges of its being cumbersome and expensive. It is only relatively so. It is never disappointing in its results, but surpasses one's expectations. It is not as much disliked by patients as acids, and it is easier to regulate the effect. Patients fear it less than knives, saws, and snares. There is little or no pain when twenty per centum solution of cocaine is used. The operation is clean, with no hæmorrhage; one can remove small exostoses and cartilaginous masses, but not large ones. A saw is better for them. Puncturing them may cause enlargement and fatty degeneration. He treats hypertrophies by puncture and incision with cautery, punctures and moves the point freely in the mass, so as to cause contraction without destroying the external tissues. His battery and various electrodes were exhibited. He has in operating used rhigoline, after Dr. Jarvis' method.

Dr. R. H. Thomas agreed with Professor Eaton's conclusions as to the general applicability of galvano-cautery, but did not consider it as successful for large hypertrophies as the cold snare. He considered twenty per centum of cocaine as far from safe for indiscriminate use. He has seen too many unpleasant effects from even weaker solutions. He never needs over ten per centum as the highest.

---

### SECTION ON PUBLIC AND INTERNATIONAL HYGIENE.

### Third Day.

The president read a paper from Dr. B. W. Richardson, of London, England, on

THE GROWTH OF PREVENTIVE MEDICINE IN GREAT BRITAIN.

Dr. Dominigos Freire, of Rio de Janeiro, Brazil, read a paper

ON VACCINATION IN YELLOW FEVER, the tenor of which was that the disease can be prevented by inoculation with attenuated virus. The paper was accompanied by microscopic specimens of the microbe of yellow fever.

Dr. Freire being asked what was his theory concerning the attenuation of the virus of yellow fever, answered, by oxidation through the red corpuscles of the blood, and the proper or improper medium into which it is placed; but, he added, all this is *as yet* only conjecture.

Dr. Bailhache, Marine Hospital Service, asked if vaccination in one family arrested the progress of the fever in that family?

Dr. Freire answered in the affirmative, stating that in families of ten and fifteen members, living in *one hut*, if vaccination was practiced after the outbreak of the fever among the members not yet affected, that this arrested the further progress of the scourge; whereas, where it was not practiced they all were stricken down with the fever and many, if not all, died.

---

### SECTION ON PSYCHOLOGICAL MEDICINE AND NERVOUS DISEASES.

### Third Day—Morning Session.

Professor Mendel, of Berlin, Prussia, read a paper on

The Origin of the Upper Facial Nerve, which was illustrated with numerous sections of the brain.

The paper was discussed by Dr. E. C. Spitzka, of New York, N. Y.

Dr. E. A. Homen, of Helsingfers, Finland, read a paper on

HISTOLOGICAL ALTERATIONS FOLLOWING AMPUTATIONS IN THE PERIPHERAL NERVES, THE SPINAL GANGLIA, AND THE MARROW.

Dr. Homen had two sets of observations, one relating to the effect on the spinal cord after amputations in dogs. In some of them he amputated the hind leg below the knee, in others the hind leg at the thigh, in others the fore leg at the shoulders. He kept some alive three weeks, some for months, and some for a year. He found that the atrophy, besides involving those limbs whose atrophy has been stated by previous observers, chiefly the posterior column, also involved a special cell-group of moderate or small-sized cells. This cell-group was atrophied when only the posterior roots were eliminated. He concluded that they had a sensory function. In most of his cases the ascending degeneration was very slight and difficult to identify. The second set of his observations related to the changes in the nerve-stumps, and corroborated those of Spitzka and others.

Dr. Homen presented some photographs illustrating the points he had stated in his paper.

Dr. E. C. Spitzka, of New York, N. Y., asked Dr. Homen more particularly as to the location of the degenerated area. He referred to the fact that in some cases a part in others the whole of the hindleg, in still others the whole of the foreleg, had been amputated ; the area of secondary ascending degeneration must have been different in each case. It seemed to him that in the last-mentioned case the degeneration should be in the comma-shaped field, in the former two in special parts of the column of Gall.

Dr. Homen replied that, on the whole, he supposed that expectation would be realized, but where he had succeeded in tearing out special posterior roots their number had been too few, and the consequent degeneration too slight, to permit of very accurate and positive location.

Dr. Otto's paper on

NUCLEUS-STAINING BY ANILINE DYES

was next read.

Dr. J. Langdon Down, of London, England, presented a paper, entitled

CASES ILLUSTRATING THE ASSOCIATION OF THE PROW-SHAPED CRANIUM WITH NEUROTIC DISEASE, and cited several interesting cases which had come under his observation.

---

THIRD DAY—AFTERNOON SESSION.

The afternoon session was almost entirely occupied in the discussion on

SYPHILIS IN ITS RELATIONS TO INSANITY.

Dr. Savage, of London, England, opened the discussion. He said there were but few new facts to add. The object of his paper was clinical, not statistical. We have to sum up experience after the syphilitic wave of fashion has passed.

His experience resembled that of most others, that severe cases of brain-syphilis follow slight evidences of secondary trouble. Local syphilitic lesions often form the starting-point of general degenerations. He was unable, in many cases, to draw any defining line between true general paralysis and degeneration following cerebral syphilis. He believes that in some cases general paralysis depends on syphilis for its origin ; but does not believe it to be anything like a universal cause. Idiocy, imbecility, and moral perversion due to congenital syphilis, he thought, was rare ; most men connected with idiot asylums consider that but few idiots have congenital syphilitic histories. Other physicians, like Dr. Bury, think a fair number of cases are due to this disease. Many syphilitic children die in utero and early life. Syphilis may produce idiocy by causing disease of the envelopes of the brain, or of the brain itself, or by causing destruction of the organs of sense. Syphilis may cause epilepsy in children, and thus lead to idiocy. Some children of syphilitic parents are morally weak, and others break down in critical periods in development. To this last class belong the children of some general paretics.

Discussion then followed upon these two points in Dr. Savage's paper.

Dr. E. D. Ferguson, of Troy, New York, instanced a case of general paralysis in a male patient who, near the time of his marriage, had contracted syphilis; which, on account of his previously correct life, was not suspected, and went on to a considerable extent before specific treatment was resorted to; when syphilis was finally suspected and treatment actually entered upon, the patient had become insane. At all times after he had contracted the disease, he had suffered from great mental depression and mortification; he could never get over the sense of shame. At the post-mortem there were found gross lesions of the membranes of the brain and of the bones of the skull.

Dr. Hurd, of Pontiac, Michigan, had no doubt but that a true syphilis will produce the simpler psychoses like mania and melancholia. He cited a case that had come under his observation, of what appeared to be acute mania, and a short time after admission it was discovered that she suffered from syphilis. She had the syphilitic rash, the rise in temperature, and these and other symptoms lasted several weeks. She was at once put upon treatment, with the effect of alleviating the symptoms, and there was a corresponding improvement in her mental condition. There was at all times, however, a tendency to relapse of the syphilitic troubles. The disease went on and displayed characteristic constitutional symptoms. After two or three years' treatment, the syphilitic trouble was finally in such a state of abeyance that the girl was sent home on trial, and has remained at home nearly a year. He had no doubt but that she would sooner or later be returned to the asylum, in consequence of the syphilitic trouble lighting up again.

Dr. C. H. Hughes, of St. Louis, Missouri, said syphilis manifests this peculiarity in the neural tissue: it produces abneural changes rather than changes within the structure of the nervous texture. This explains the facility with which syphilitic mental aberration is removable under adequate and vigorous specific treatment. He had seen syphilis often remain without manifestation for years after insanity, and finally develop in locomotor ataxy. We know there is such a thing as syphilophobia, and that it may exist without the pre-existence of any syphilitic poisoning. Syphilitic general paralysis, he had no doubt, was exceedingly common as the result of this poison.

Dr. W. W. Godding, of Washington, D. C., said he had never seen any mental symptoms whereby he could diagnose a case as syphilitic insanity. He cited the interesting case of a girl in his hospital, who came to him about nine months since in the most advanced stage of dementia. Syphilis was only suspected after she had been in the hospital some time, and had remained profoundly depressed. She was placed at once upon specific treatment, with the effect of bringing about an immediate change for the better, and she is to-day in a very comfortable condition of bodily and mental health.

Dr. E. C. Spitzka, of New York, N. Y., referred to a febrile condition in secondary syphilis, which is complicated with acute delirious mental disturbance. This he believed was a very rare thing. He had seen one case seven years ago, and had not seen anything written upon the subject since that time. The discovery had been made by Finger that during the roseola there was abolition of the knee-jerk. Finger had reported sixty-one cases in which the knee-jerk was absent.

Dr. E. N. Brush, of Philadelphia, Pennsylvania, said that two cases of syphilis were now under his observation, which served to confirm the statement of Finger. One of these cases had a short time ago a remission of symptoms of acutely maniacal excitement. He had been quiet and coherent, had parole of the grounds. For five days was very much excited, and there was a slight return of the knee-reflex, which had previously been entirely absent. He tore his clothing, had hallucinations, and disturbances of sight and hearing. Dr. Brush did not believe we should adopt the term syphilitic insanity.

Dr. T. W. Fisher, of Boston, Massachusetts, cited the case of an officer in one of the hospitals at Boston, who contracted syphilis, and suffered from a great sense of mortification, and went into a state of apprehension and sleeplessness, and finally developed delusions of suspicion. Under asylum treatment he recovered after the syphilitic symptoms had disappeared.

Dr. Brown, of Barre, Massachusetts, was glad to be able to support the ground taken by Drs. Savage and Down, in reference to very few imbecile children having congenital syphilitic histories. In his experience of thirty-five years he had noticed but one and one-half per centum.

Dr. Savage said that he agreed with Dr. Godding, that there was no such thing as syphilitic insanity. One might speak of a specific cause of asthma, and give it a title accordingly. The less said about syphilitic insanity the better in his opinion. As to the cases recorded of the febrile disturbance of constitutional syphilis, he was glad to note that it was, after all, not so uncommon, although it had hitherto been hardly recognized.

Dr. Savage then proceeded to the next branch of the discussion,

INSANITY DUE TO ACUTE SYPHILIS.

Such cases are rare. Probably some acute cases follow the delirium which may follow with the rash. Many lunatics exhibit venereal diseases contracted while insane. Insanity may follow the onset of secondary symptoms of the more severe type—thus, with optic neuritis, with ulcerations about the face—or it may appear with the onset of local paralysis of the third or other cranial nerve. It may occur with the neuralgia or sleeplessness of syphilis. The disfigurement may cause insanity. There are cases in which syphilis has acted as a moral cause and has set up a form of hypochondriasis.

Dr. Savage next passed to

SYPHILIS PRODUCING EPILEPSY, WITH OR WITHOUT INSANITY.

The epilepsy may be the chief symptom or only a sign of widely spread disease, or it may be a symptom of general paralysis, or of locomotor ataxy. He said cases of epilepsy had come under his observation with a syphilitic history, in which he had not been able to find coarse lesions, as had been claimed to exist by Ferrier and Hitzig.

Dr. Hughes asked Dr. Savage if in his post-mortems he had been unable to find foci, either vaso-motor or vascular changes, microscopically.

Dr. Savage answered that the arterial disease was so general that he had not been able definitely to say that on the left side there was more arteritis than on the right.

Dr. Savage also said there was a good group of cases in which

PROGRESSIVE WEAK-MINDEDNESS

follows constitutional syphilis. It may be preceded by some motor symptoms, by either the ocular motor trouble, a monoplegia or hemiplegia, in one by aphasia, and in another with other motor trouble. There was another group in which sensory trouble, some temporary loss of vision or taste or hearing, some aphasia, which may be purely muscular, or some temporary giddiness may be the first symptom, which is followed by progressive degeneration, which is not of the same kind and not to be grouped with true general paralysis of the insane depending upon syphilis.

Dr. Channing asked Dr. Savage how he would classify the cases just described.

Dr. Savage replied that he would classify them as organic dementia; that seemed as good a classification as any.

Dr. W. Channing, of Boston, Massachusetts, asked if the term syphilitic dementia could be used, if there were sufficient cases to justify it.

Dr. Savage preferred organic dementia of syphilitic origin.

Dr. Gundry next discussed the question, and cited the case of a most respectable and highly connected young lady whose friends had noticed in her a growing tendency to *double entendres*, to slight smuttiness in conversation, and who were at a loss to account for this. He suspected syphilis, but was not for a long time listened to. Finally, after getting the patient on specific treatment, these morbid peculiarities disappeared.

Dr. Savage next discussed

THE GENERAL PATHOLOGY OF CASES OF INSANITY WITH SYPHILIS.

There may be a moral element in some cases, this being of two kinds, either producing hypochondriasis or true melancholia. Syphilitic cachexia may cause melancholic symptoms. Disease of the vessels, small or great, may produce different sets of symptoms.

Gummata are not so common as one would have expected. Inflammation of the membranes in both young and old may cause disease, such as idiocy or acute insanity.

Whether there are any changes in the neuroglia is doubtful. There are changes produced in the nutrition of the various parts, leaving a form of weakness which may act as a cause of fresh changes that may run an acute or chronic course.

Dr. Spitzka's experience coincided with that of Dr. Savage as to the absence of gummata.

He corroborated the conclusions of Dr. Savage. The connection between syphilis and tabes dorsalis was still a mooted question. He gave the case of an actor who had been under his observation for some time. This patient has abolition of both knee-jerks. His disease was at times controlled by treatment, and then recurred. He had had in the course of one day fifty or sixty attacks of a peculiar kind of *petit mal*, in which he lost consciousness for a moment while coming upon the stage oftentimes, but so briefly that he could recollect himself. In one case, where he had to cross a foot-bridge, he had an attack in the course of passing over, but went on as if nothing had happened, the *petit mal* losing its character as a loss of consciousness and becoming replaced by a peculiar sensory disturbance. He found that, accompanied by the prodromal feeling, all the faces of the audience were exactly the same. This sensation passed like an electric flash. He still has these peculiarities, although they are becoming rarer. They are a matter of interest to the patient himself, and he observes their course and peculiar symptoms.

After the reading of a paper by Dr. Ingram, of Washington, D. C., on

GUNSHOT WOUND OF THE SPINAL CORD, the section adjourned until Thursday at 11 A. M.

### GENERAL MEETING.

### FOURTH DAY.

The congress convened at 10 A. M.

Dr. A. L. Gihon, U. S. navy, offered the following resolution, which was adopted:

*Resolved*, That the President of the Congress be authorized to appoint a committee, to consist of an equal number of members from each nationality represented in the Congress, for the purpose of selecting the place of meeting of the Tenth International Medical Congress, to be held in the year 1890; which committee shall report on Friday morning, immediately before the address of Dr. Brandford.

The following committee was appointed to name the next place of meeting of the congress:

Argentine Republic—Dr. Villa, of Buenos Ayres.

Austria-Hungary—Dr. Farkas, of Buda-Pesth.
Belgium—Dr. Servais, of Antwerp.
Brazil—Dr. Freire, of Rio de Janeiro.
China—Dr. Boone, of Shanghai.
Egypt—Dr. Grant, Bey, of Cairo.
France—Dr. Landolt, of Paris.
German Empire—Dr. Martin, of Berlin.
Great Britain—Dr. Pavy, of London.
Italy—Dr. Semmola, of Naples.
Japan—Dr. Saigo, of Imperial Navy.
Mexico—Dr. Alvarado, of Mexico.
Roumania—Dr. Monolescu, of Bucharest.
Russia—Dr. Reyher, of St. Petersburg.
Spain—Dr. Lalearda, of Seville.
Sweden and Norway—Dr. Tillman, of Halinstadt.
Switzerland—Dr. Cordes, of Geneva.
Turkey—Dr. Post, of Beirút.
United States—Dr. Gihon, United States Navy.

---

Dr. P. G. Unna, of Hamburg, Germany, then delivered a general address on the

RELATIONS OF DERMATOLOGY TO GENERAL MEDICINE.

He endeavored to prove that every general practitioner has the greatest interest that dermatology should be more deeply and extensively studied. Dermatology, as a special branch, is still young, and not yet past the state of formation. The immense difficulties in the way of the study of diseases of the skin, which have up to this time disturbed its continuous development, are in great part well founded, as well in the external position of this organ as in its complicated structure. The author demonstrated these complications in detail, showed the differences in the appearance of the symptoms according to regional differences of the skin, the changes in symptoms in the course of the development of skin diseases, the varieties produced by external agents, the influence of climate, season, nature of countries, of races, sex and age. Among the external agents the parasites, according to our present knowledge, occupy the most prominent place.

These difficulties will in future be overcome only by minute analysis of each of the several symptoms of skin diseases. All progress in this direction will be of the greatest value for general pathology and therapeutics, because these facts are proved in the human tissue by means of the naked eye. Dermatology, studied properly, will advance all other parts of medicine—internal medicine as well as surgery—occupying a middle ground between them. Dr. Unna recommends, in place of experiments on animals for pathological and therapeutical experiments, to use the human skin, and showed by several examples how this method has led already to the knowledge of new facts. The endowment of new chairs and of separate private laboratories is not sufficient for a thorough investigation of skin diseases. He recommends the establishment of a central institute, where noted scientists could work together, where all means and methods of study could be condensed. Then dermatology can be raised to the rank of one of our most important specialties in medicine, and contribute largely to the progress of knowledge in all branches. Finally, he expressed the hope that the United States, always so liberal in the promotion of science, will be the first to develop this ideal.

---

### SECTION ON GENERAL MEDICINE.

---

FOURTH DAY—MORNING SESSION.

Dr. Ephraim Cutter, of New York, N. Y., read a paper entitled

MORPHOLOGY OF RHEUMATIC BLOOD, and illustrated it with stereopticon slides. He attributed the increased force of cardiac, with consequent exhaustion and inflammation, to the great adhesiveness of the blood.

Professor Mariano Semmola, of Naples, Italy, read a pager on the

PATHOGENESIS OF ALBUMINURIA.

He gave the results of original investigations made at the University of Naples.

Dr. Nunn discussed the paper, speaking favorably of the albumen diet.

Dr. Cutter, of New York, N. Y., regarded the diagnosis of Bright's disease as imperfect unless there are albumen and casts.

NOTES ON THE TREATMENT OF PHTHISIS, MORE PARTICULARLY THAT BY INTRA-PULMONARY INJECTION.

R. Singleton Smith, M. D., Bristol, England, read a paper on this subject. Since the last International Congress at Copenhagen, in 1884, numerous attempts have been made to do more than had previously been attempted for a disease in which the *vis medicatrix naturæ* does so little. The reader of this paper briefly summed up the various methods which have been recently suggested since the discovery of the bacillus of tubercle, and expressed his belief in the possibility of benefit by treatment directed toward the destruction of the bacillary growth. He reported the result of his experience with regard to gaseous rectal injections, and said that in consequence of the absence of all indications of benefit he had given up this method entirely. He alluded to the work done by Professor Pepper, of Philadelphia, Pennsylvania, and Drs. Beverly Robinson and White, of New York, N. Y. In carrying out the method of intra-pulmonary injections suggested by these and other workers, he had met with partial success in a series of cases reported in the *British Medical Journal* in 1886.

In consequence of the proved utility of iodoform in chest disease, as shown by a steadily increasing mass of evidence since its first introduction for this purpose by Professor Semmola, in 1878, and supported by a series of cases presented to the International Congress of 1884, in which the author found increase of weight, improved appetite, diminution of temperature, and general improvement, under the administration of iodoform given by the alimentary canal, it was thought that iodoform would be the best substance to employ for injection into the parenchyma of the lung. The clinical utility of iodoform being the ground on which its use for this purpose has been founded, the evidence is not shaken by any statistics as to the comparatively feeble powers of the drug as a germicide.

The insolubility of the drug is the chief difficulty; various solvents have been used, but with only partial success. Ether is objectionable, because of its effects on the brain; giddiness and other feelings of discomfort rather alarm the patient, and give rise to an unwillingness to have a frequent repetition of the injections. Eucalyptus oil is irritating; two cases were mentioned in which acute pleuritis, with much pain, rise of temperature, and effusion had followed the injection of an iodoform solution in oil of eucalyptus. The vaseline oil, either alone or in combination with eucalyptol, had also been used, but the author considers the question, what is the best fluid to inject, to be still unsolved. He did not advocate the use of solutions containing free iodine or bichloride of mercury, and he would not in future employ any fluid for injection into lung which had not previously been tested hypodermically; if it gave rise to much inflammatory irritation in the subcutaneous cellular tissue he would not venture to inject it into the lung. He was of opinion that injections into cellular tissue might possibly be of some little service, although there was as yet not much reliable evidence on this point, but they would serve as a reliable test whether any given fluid was suitable for deep intra-pulmonary injection.

It is true that if iodoform be of use, as the clinical evidence indicates, then it is likely to be of far greater utility when injected, even in small quantity, into the focus of a diseased patch than when given in larger doses diffused throughout the whole body. Such injections have been shown to be not especially hazardous. Even the cases in which pleuritis has occurred had recovered completely from the attack in the course of a few days, and possibly the pleuritis was due to the failure of the fluid to pass beyond the pleural cavity into the lung-substance. Nevertheless, the author would not advocate the use of such injections in cases which were hopeless, neither would he employ them in cases where other and less active measures were accomplishing the object in view. He concluded his paper by urging perseverence in spite of failure, and by expressing his belief that what as yet was only a tentative investigation would ultimately result in numerous and signal successes.

Dr. Truax, of New York, said that he had little faith in iodoform, as he had cultivated the bacillus tuberculosis in its solution.

---

### Afternoon Session.

Dr. Pavy, of London, England, spoke on

### DIABETES.

This disease has always been regarded as an inscrutable one. There are still many points open for earnest study and patient investigation. The nature of the disease is that of faulty disposal and assimilation of food elements. Foods taken into the body are classed as nitrogenous, fatty, and the carbohydrates. It is this latter class that especially concern us in diabetes. Starch-dextrine, lactic and cane sugar go to make up this group, and each has an equal action, when ingested, in causing the condition of diabetes.

Normally the carbohydrates are disposed of in the portal vein, whence they are carried to the liver and there assimilated. Experiments in which defibrinated blood or oxygen have been injected into the portal vein have been followed by marked evidences of sugar in the urine. Similar results have been produced in animals by forced respiration, thereby supersaturating the blood with oxygen. Vaso-motor paralysis of the hepatic vessels, by causing an excess of blood, prevents the proper deoxygenation of the blood, and similarly causes sugar to be present in the urine. This condition allows the carbohydrates to be converted into sugar and pass through into the general circulation.

In the celebrated experiments of Bernard, in puncturing the floor of the fourth ventricle, he also noticed a vaso-motor paralysis of the hepatic vessels. It is noticeable that in those cases of vaso-motor paralysis of the chylopoiëtic system where there is a red tongue the disease is more severe, undoubtedly owing to the fact that the paralysis has extended more generally throughout the circulatory system.

In health there is only a faint trace of sugar in the blood and the urine. The latter is always a reliable index as to the amount of sugar in the blood. In diabetes the sugar reaches the blood directly, without going through the process of assimilation in the liver. In health, sugar is stopped before it enters into the general circulation, but in diabetes it is found in the blood directly in proportion to the quantity of carbohydrates taken. Sometimes even in health there is sugar in considerable quantity in the urine, in cases where there has been taken very large quantities of the carbohydrates, showing that there is a normal limit to the assimilative power of the liver, which, if exceeded, will result in a diabetic condition. The liver is a fat, rather than a sugar-producing organ, converting animal starch into sugar and then into fat. The liver is different from other organs in that it has small arteries and large veins. The contents of the portal vein should be in a decidedly venous condition, otherwise we will get sugar into the general circulation.

Diabetes is of a neurotic origin, and it is well established that nervous conditions influence in a material manner the condition of the patient.

In considering these cases the first thing to do is to test for sugar in the urine, and in order that we may fully appreciate the progress of the case this test should be a quantitative one.

In selecting urine for examination it is necessary to procure both an evening and a morning specimen. It frequently occurs that sugar is only present directly after the taking into the body of carbohydrate foods. It is owing to the fact that specimens of urine are procured at different times and after different conditions of diet that physicians differ so often in the diagnosis of diabetes.

Among the various tests for sugar probably the most accurate is the copper test, in the form of Fehling's solution. The great objection is that this solution will throw down a precipitate on boiling, which is very liable to mask the test. In order to obviate this I have had pellets made of the solid ingredients of this solution, in an anhydrous form, such as can be at any time dissolved in water for use.

When patients come to us we should not ask them, but tell them, what they have been eating or drinking. This can be accurately done by the quantitative test. This is best and most easily accomplished by the decolorization test, which is done in the following way :

At the bottom of a long graduated pipette is attached a tube running into a flask containing a given quantity of a solution of sulphate of copper, differing from Fehling's solution only in containing ammonia and potash instead of the latter alone (in order to prevent the precipitation of the oxide); at the upper part of the flask is placed an escape-tube. At the rubber tube through which the urine escapes into the flask is placed a compressor controlled by a screw, which regulates perfectly the flow of the urine. After the copper solution is brought to a boiling point, by a flame placed underneath the flask, the urine is allowed to enter the flask, drop by drop, until the copper solution is exactly decolorized. By reading upon the graduated pipette the amount of the urine which has been necessary to decolorize the known quantity of copper solution, we can form a proportion by which an accurate estimate can be made of the contained sugar. By this means we can follow the course of the disease even more accurately than the clinician who, with his stethoscope, watches, from day to day, the progress or retrocession of pulmonary disease.

It is well to note, in passing, that in diabetes we sometimes find albumen in the urine, and this is often present just in proportion as the patient improves or gets worse. A test for albumen, which has no known fallacy, is that with citric acid and the ferrocyanide of sodium. The objection to the nitric-acid test is that it may precipitate either uric acid or the oleo-resins. In the above test, if a pellet of citric acid is dissolved in the urine and boiled, we may then get a precipitate of uric acid, but upon the addition of the ferrocyanide of sodium the solution invariably clears up unless albumen be present.

In considering the disease itself, we find it has different grades and intensity. The liver, in health, has not unlimited control in the assimilative power over the carbohydrates, and diabetes may be said to exist when that power is below the ordinary. When a person first comes to have diabetes, as we have stated, it is through faulty assimilation; it does occur, however, that as the disease progresses, the sugar is formed from the tissues themselves, as is proved by its constant presence when the patient is upon a strictly meat diet.

Age influences greatly the occurrence of the disease. Out of 1,360 cases tabulated, forty-five per cent. occurred between forty and sixty years of age. Age also affects prognosis ; young subjects rarely recover, the disease ordinarily terminating in two years. In old people the prognosis is more favorable, many recovering. One reason, possibly, for the tendency of children to do badly, is that they take unsatisfactory care of themselves, while elderly persons are oppositely inclined.

In treating young subjects we must try merely to stay the disease ; we cannot stop it. We take away the sugar, the patient temporarily improves, and believes himself cured. He is borne up only by false hopes.

In young subjects it seems to have a progressive character, not unlike progressive muscular atrophy, or of loco-motor ataxia.

For elderly patients we can do much. Appropriate diet is absolutely essential.

Dr. Herrick, of Cleveland, Ohio, asked whether Dr. Pavy considered the condition of the urine the objective point in the treatment, or did he aim his treatment directly to the abnormal condition in the portal and hepatic circulation.

Dr. Pavy said that he looked upon the lessening of sugar as an evidence that the assimilative power of the carbo-hydrate is improved The urine is an index of sugar plus the power within.

Dr. Truax, of New York, N. Y., asked if Dr. Pavy had found where acetic ether was present that death followed quicker.

Dr. Pavy replied that he was not a believer in acetonæmia.

Dr. Stockman read a paper for Dr. R. W. Phillips, of Edinburgh, Scotland, entitled

THE ETIOLOGY OF PHTHISIS.

He claimed that experiments go to show that ptomaines are the principal etiological factor in phthisis. In experiments where the ptomaines have been injected the animals give all the characteristic symptoms of phthisis, such as sweating, great depression, etc.

Dr. Phillips sent with his paper a tabulated report of experiments with injections of atropine, which he claims counteracts the action of the ptomaines.

Dr. Herrick, of Cleveland, Ohio, said that he believed that the deposits in the lung were excretions from the blood of material which the natural eliminatives were unable to dispose of. He did not believe the bacillus tuberculosis to be the cause of phthisis *ipso facto*.

Dr. Whitmarsh believed that a very large number of the race have tuberculosis ; it often lies dormant, yet the germ is there, like fuel, only needing a match to set it on fire. The early stage of phthisis is curable, but unfortunately that stage is too often overlooked.

Professor Arnold, the President, thought that Koch still had the best side of the argument. The crucial experiment has been made, that when inoculations are made with sputa containing the bacillus tuberculosis, we get phthisis, but do not get phthisis under any other circumstances.

Dr. Truax, of New York, N. Y., called attention to the additional evidence of the power in the bacillus tuberculosis, viz., that repeated cultures of the germ still retain the power to cause phthisis.

The Secretary read a paper by Dr. W. B. Neftel, entitled

SOME CONSIDERATIONS ON THE PATHOGENESIS OF DISEASES OF WOMEN.

He endeavors to prove, by experiments upon the lower animals, that compression of the chest will cause phthisis. Although his experiments did not prove this, they did show that they would cause albuminuria, which he attributed to venous stasis.

From these experiments he made analogous comparisons of the deleterious effects of wearing corsets and high-heeled shoes.

He suggested that the Congress appoint a commission to investigate the various modes of feminine dress of the different nationalities, and select such forms as are both practical and useful. He thought that in this way, with the sanction of the profession, some impression might be made upon the female sex to change their present mode of dress.

Dr. Herrick thought that experiments upon the lower animals were of little use in forming opinions of disease in the human subject.

Dr. Price, New York, N. Y., agreed with Dr. Herrick in the main. He did not think that bacilli were the cause of phthisis.

Dr. Finlay, Mansfield, Ohio, asked if statistics showed that the difference in dress caused a corresponding increase of mortality among women.

Dr. John Ege, Reading, Pennsylvania, thought it very necessary that each air-cell should have free play in performing its function, otherwise it would be a favorable soil in which the bacillus could grow. He believes in the germ-theory of phthisis.

---

### SECTION ON GENERAL SURGERY.

### FOURTH DAY—MORNING SESSION.

Dr. Burney read for Dr. Richardson, of St.

Louis, Missouri, a report of a case of gastrotomy for the relief of foreign bodies in the throat. The speaker also mentioned a case in which he himself had operated, a man having swallowed an ordinary plated case-knife, which he presented at the meeting. While playing with his family the knife slipped from his fingers as he pretended to swallow it, and really did. The writer did not believe in the continued suture in this operation, but the interrupted, one and one-eighth of an inch apart.

In discussing Professor Dennis' paper, Dr. Myers remarked that MacEwen used only digital pressure in the amputation of the thigh-joint.

Professor MacLean, of Detroit, Michigan, said that Dr. Dennis' method was the principal point in the paper; he had had the best results in Lister's abdominal compressor.

Dr. Weeks, of Maine, stated that he used the ordinary rubber-bandage with a compress, the bandage being four thicknesses and secured much the same as Dr. Dennis'.

Dr. Manly, of New York, N. Y., thought it difficult to find anything to take the place of Lister's compressor.

Dr. Reyher, of St. Petersburg, Russia, said he had had some unpleasant experiences with Listerism. He was in the habit of first excising the head of the bone, then piercing the thigh and ligating in two parts, then amputating the legs; the flaps were then under the control of the surgeon, and he could work at leisure, but he usually put a rubber-bandage above.

Dr. N. Smith thought that the less tissue left the better, and that he was satisfied with Brodie's operation, the anterior and posterior skin-flaps; as for controlling hæmorrhage, the hands were best; he had not had much experience with the rectal staff.

Dr. Springler, of Dresden, Saxony, remarked that about one-fifth of these cases healed primarily; one-fifth in about three weeks, and one-fifth in about two to three years, and then perhaps would die from the amputation and the prolonged drain on the system.

In discussing aspiration of the hip-joint, Dr. Lange, of New York, stated he thought the conclusions on the subject were rather premature.

The question was asked if the fluid were examined under the microscope for bacilli; also whether the fluid was removed as a curative measure or only to relieve pain.

To the former question Mr. Owen replied it was not, and as to the latter it was both; replying that if you are in doubt as to these cases, put in your trocar and cannula; you can do no harm and may do good.

In regard to Dr. Garmody's paper, Dr. Burney stated he had a case of a boy whose skull was fractured, and who shortly afterward was taken with maniacal attacks of screaming epilepsy. He was trephined and two buttons of bone were removed; a second operation was performed and the dura mater incised, when immediately a large quantity of cerebro-spinal fluid gushed out, which very much alarmed the speaker; sponges were quickly placed in the cavity: subsequently the wound was sewed up. Since that time he has gradually improved.

Dr. Manley, of New York, N. Y., read a paper on a case of

GUN-SHOT WOUND OF THE LARGE INTESTINE, WITH A SUCCESSFUL RESULT BY LAPAROTOMY.

The patient was operated on twice. The speaker considered that opening of the peritoneal cavity of a man was far different to opening that of a woman, and liable to more risks; the breathing of a man is largely abdominal, while that of a women is almost entirely thoracic; the incision in the man should not be a bit larger than is necessary, because of his laborious work being liable to produce ventral hernia after recovery. For exploratory operations he made the median incision, but thought there might be exceptions to this rule. In regard to the drainage-tube in these gun-shot wounds of the abdomen, he thought there was no need of them when the peritoneal cavity was healthy and laparotomy was performed soon after the injury, and although he had put one in, in the case spoken of, he should not do it in the next one. Of course there might be exceptions, but he belived the tube was a source of irritation and more liable to produce hernia.

---

AFTERNOON SESSION.

Dr. Robert Newman, of New York, read a paper on

THE USE OF THE GALVANO-CAUTERY SOUND, particularly in hypertrophy of the prostate gland, with report of cases. The frequency of the application of the cautery, he stated, would depend much on the condition of the patient, but it averaged about from every three to six days ; the cautery was not to be red hot, but simply to show a bright light. The apparatus was exhibited to the section. The advantages claimed are that there is no hæmorrhage, the healing is more rapid, and there is no septicæmia. Out of these cases 91 per centum are made comfortable and given a new lease of life.

Dr. Carnochan, of New York, N. Y., presented a rare specimen of

BONY UNION OF THE NECK OF THE FEMUR WITHIN THE CAPSULE, after fracture, occurring in a woman seventy years of age. As she had plenty of means she had every attention. She was kept in her fracture-bed for nine months, rest being the method of treatment. The value of the specimen was very great, especially in courts of law.

Dr. F. Lemoyne, of Pittsburg, Pennsylvania, presented some drawings of an united fracture of the femur, and history of a successful case in which he had applied his method of treatment, viz.,

DOUBLE SPLICE AND WIRED CLAMPS.

The treatment has been successful in three cases, two of the humerus and this one of the femur. The bone was cut down upon and the fragments found to be overlapping nearly three inches. The ends were sawed off, the upper fragment in the form of a wedge; from the lower one a wedge-shaped piece of bone was removed, so that the upper fragment fitted into it. A hole was drilled in both bones about one and a half inches from the fractured ends. A stiff, flat steel bar, with a prong at either end, was placed lengthwise on the surface of the bone, so that the prongs were inserted into the holes; a stout piece of wire was then passed around the clamp and femur at either end, and twisted close up, holding the clamp in position. The wires and clamp remained there nine weeks, when the wound was reopened and the clamp removed. The bone was firmly united, but the limb is two and a half inches shorter.

Dr. Manley, of New York, N. Y., stated that he had had considerable experience in wiring bones, and thought the bones when lapping very much should be wired at the first; he always left the wires in, as they did no harm. In one case of compound comminuted fracture he had followed this method and secured perfect union.

Dr. Lemoyne remarked, he thought that his method was original, everything else had failed in the case of one of those reported who had undergone a number of operations previously. In all of his cases so treated he had met with success; he did not believe in wiring in primary fractures.

Dr. Gibson exhibited some wire extension splints for fractures at or near the joints; he had used them since 1879. They were a modification of the wire splint of Dr. Nathan R. Smith.

Dr. Meyers remarked that he thought the day of extension, except in compound fractures, had gone by; he was very well satisfied with plaster-of-Paris board, or some simple and light dressing.

Dr. Gibson replied that he wished it understood that he did not use this splint in all cases, but for those fractures at or near the joints.

In discussing Dr. Manley's paper, Dr. Quimby, of Jersey City, New Jersey, stated he thought in many cases of wounds the tubes were left in too long. He quoted one case of laparotomy in which no tubes were introduced. Death followed the operation; the wound was reopened, and the peritoneal cavity found perfectly dry.

Dr. Manley concluded by saying that in ovariotomy, where substances were removed, it was different, but in gunshot wounds he considered the tubes injurious.

